# Draft Genome of Akame (*Lates Japonicus*) Reveals Possible Genetic Mechanisms for Long-Term Persistence and Adaptive Evolution with Low Genetic Diversity

**DOI:** 10.1093/gbe/evae174

**Published:** 2024-08-07

**Authors:** Yasuyuki Hashiguchi, Tappei Mishina, Hirohiko Takeshima, Kouji Nakayama, Hideaki Tanoue, Naohiko Takeshita, Hiroshi Takahashi

**Affiliations:** Department of Biology, Faculty of Medicine, Osaka Medical and Pharmaceutical University, Takatsuki, Osaka 569-0801, Japan; Laboratory for Chromosome Segregation, RIKEN Center for Biosystems Dynamics Research (BDR), Chuo-ku, Kobe 650-0047, Japan; Faculty of Agriculture, Kyushu University, Nishi-ku, Fukuoka 819-0395, Japan; Faculty of Marine Bioscience, Research Center for Marine Biosciences, Fukui Prefectural University, Obama, Fukui 917-0003, Japan; Division of Applied Biosciences, Graduate School of Agriculture, Kyoto University, Kyoto 606-8502, Japan; Operations Evaluation Division, General Planning and Coordination Department, Headquarters, Japan Fisheries Research and Education Agency, Yokohama, Kanagawa 221-8529, Japan; Department of Applied Aquabiology, National Fisheries University, Shimonoseki, Yamaguchi 759-6595, Japan; Department of Applied Aquabiology, National Fisheries University, Shimonoseki, Yamaguchi 759-6595, Japan

**Keywords:** akame, draft genome, inbreeding depression, genetic diversity, genetic load, balancing selection

## Abstract

It is known that some endangered species have persisted for thousands of years despite their very small effective population sizes and low levels of genetic polymorphisms. To understand the genetic mechanisms of long-term persistence in threatened species, we determined the whole genome sequences of akame (*Lates japonicus*), which has survived for a long time with extremely low genetic variations. Genome-wide heterozygosity in akame was estimated to be 3.3 to 3.4 × 10^−4^/bp, one of the smallest values in teleost fishes. Analysis of demographic history revealed that the effective population size in akame was around 1,000 from 30,000 years ago to the recent past. The relatively high ratio of nonsynonymous to synonymous heterozygosity in akame indicated an increased genetic load. However, a detailed analysis of genetic diversity in the akame genome revealed that multiple genomic regions, including genes involved in immunity, synaptic development, and olfactory sensory systems, have retained relatively high nucleotide polymorphisms. This implies that the akame genome has preserved the functional genetic variations by balancing selection, to avoid a reduction in viability and loss of adaptive potential. Analysis of synonymous and nonsynonymous nucleotide substitution rates has detected signs of positive selection in many akame genes, suggesting adaptive evolution to temperate waters after the speciation of akame and its close relative, barramundi (*Lates calcarifer*). Our results indicate that the functional genetic diversity likely contributed to the long-term persistence of this species by avoiding the harmful effects of the population size reduction.

**Significance**

Population size decline and loss of genetic diversity in threatened species have been considered to increase the risk of extinction. However, akame is known to have persisted for thousands of years despite low genetic diversity. In this study, we sequenced the draft genome of akame and analyzed its genomic diversity. In akame, heterozygosity of single nucleotide variants was one of the lowest in teleost fishes. However, several genomic regions in the akame genome showed high levels of polymorphism. Our results indicate that, in akame, some functional genetic variations have been preserved by selective forces, to avoid a reduction in viability and loss of adaptive potential.

## Introduction

In naturally outbreeding organisms, the decrease in genetic diversity owing to a reduction in population size can result in a decline of mean phenotypic values across various fitness-related traits, thereby limiting the evolutionary potential of the population. Population size reduction also results in the accumulation of deleterious genetic variants (i.e. genetic load) due to the dominance of genetic drift over purifying selection and the elevated level of inbreeding. Consequently, population decline in threatened species has been considered to increase the risk of extinction in the wild (Frankham et al. 2010; Allendorf et al. 2013). Indeed, the negative effects of population size reduction such as inbreeding depression, loss of adaptive potential, and elevated genetic load, have been documented in numerous endangered wild species that have experienced severe population decline and low genetic diversity (Frankham et al. 2010; Allendorf et al. 2013; de Villemereuil et al. 2019; Khan et al. 2021).

However, it is also known that certain endangered species are able to sustain their populations for extended periods of time, despite experiencing population declines and reduction in genetic diversity. For instance, the eastern subspecies of mountain gorilla *Gorilla beringei beringei* has undergone a prolonged population decline over 100,000 years (Xue et al. 2015). Similarly, certain island populations of channel island fox *Urocyon littoralis*, have persisted for thousands of years despite extremely small population size (Robinson et al. 2016, 2018). Conversely, some species, such as the western lowland gorilla *Gorilla gorilla* and Sumatran orangutan *Pongo abelii*, are now highly endangered despite maintaining relatively high genetic diversity (Robinson et al. 2016; Teixeira and Huber 2021). Thus, a traditionally accepted concept in conservation genetics that the extinction risk of a species can be assessed in terms of genetic diversity or effective population size (*N*_e_), has become controversial (Teixeira and Huber 2021; Mathur et al. 2023; Wilder et al. 2023).

Akame, also known as the Japanese lates (*Lates japonicus*), is a rare and endangered teleost fish species distributed on the Pacific coast of Western Japan, primarily in Kochi and Miyazaki Prefectures (Iwatsuki et al. 1993). Akame is considered to be a notable example of a species that has persisted for an extended periods with a very small population size. Our previous analyses utilizing mtDNA, AFLP, and ddRAD-Seq markers have indicated that the level of genetic diversity in akame is comparable to that observed in some seriously endangered freshwater fishes that exhibit the lowest genetic diversity among vertebrates (Takahashi et al. 2015; Naito et al. 2023). If the long-term persistence in akame is not merely a result of chance, it is likely that akame possesses genetic mechanisms to avoid adverse effects resulting from increased genetic drift and inbreeding, such as (i) purging deleterious recessive alleles by natural selection (Crnokrak and Barrett 2002; Larsen et al. 2011; Robinson et al. 2018; Khan et al. 2021); (ii) maintaining genetic diversity in specific genomic regions that require high polymorphism, such as those containing genes associated with the immune system, through strong selective pressure (Hedrick 2002; Alonso et al. 2008); and (iii) utilizing phenotypic plasticity through epigenetic regulation (Vergeer et al. 2012). Thus, conducting a genome-wide analysis of the genetic diversity in akame would yield valuable insights into the genetic mechanisms, preventing the harmful effects of inbreeding and the loss of adaptive potential in species with low genetic diversity. Moreover, by providing empirical data on both neutral and functional genetic variations, studies on the genetic mechanisms of long-term persistence in akame are likely to contribute to a recent debate on the relative importance of neutral and functional genetic variations in the assessment of extinction risk (Kardos et al. 2021; Teixeira and Huber 2021).

Akame has a wide-ranging and abundant counterpart, the barramundi or Asian seabass (*Lates calcarifer*). There are notable biological and ecological differences between akame and barramundi. Akame is the only species of the genus *Lates* in temperate regions (Katayama and Taki 1984; Iwatsuki et al. 1993) and does not enter freshwater environments throughout its life history (Iwatsuki et al. 1993; Gonzalvo et al. 2021). By contrast, barramundi is a tropical marine species that is widely distributed in the Asia-Pacific region, inhabiting coastal marine and estuarine to freshwater habitats (Larson 1999). Genome-wide genetic diversity in barramundi is known to be relatively high (Vij et al. 2016), similar to other marine teleost fishes (Jones et al. 2012; Wu et al. 2014; Barry et al. 2021). A chromosome-level genome assembly has been published for barramundi (Vij et al. 2016) and thus, comparative genomics analysis of akame and barramundi may elucidate the impact of population size reduction on the genetic diversity, genetic load, and adaptive potential in akame populations.

**Fig. 1.**
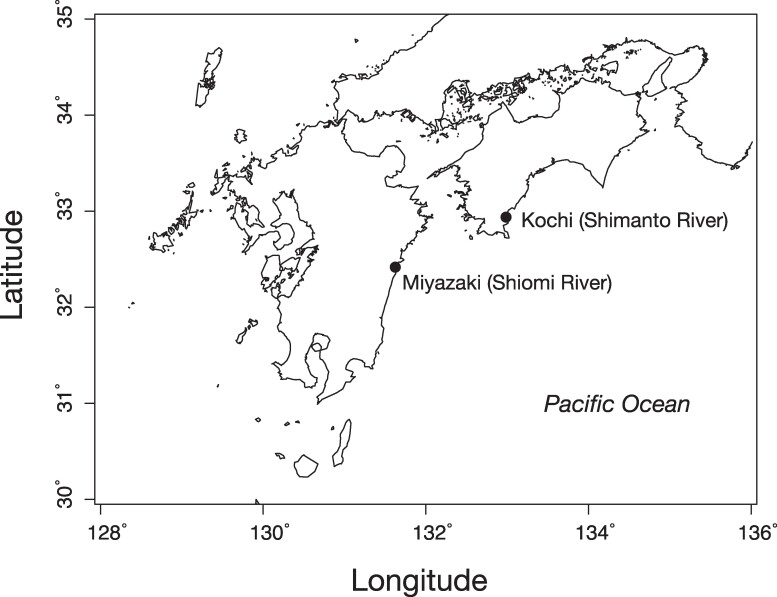
Sampling locations of the two akame individuals (Kochi and Miyazaki) used in this study.

In the present study, we sequenced the draft genomes of two akame individuals collected from the Kochi and Miyazaki regions in southwestern Japan (Fig. 1), to understand the genetic mechanisms involved in avoiding the adverse effects of low genetic diversity in wild organisms. Analysis of genetic diversity and historical population demography showed that akame has persisted from 30,000 years ago to the recent past with a very small effective population size. We also analyzed the genomic distribution of the neutral and functional genetic variations in akame, to assess the relative importance of the genome-wide genetic diversity for the long-term persistence of this species. In addition, we compared the evolutionary rates of akame and barramundi genes to infer the efficacy of selection in akame after the speciation of the two species. Our results indicate that wild populations of akame have persisted for extended periods under selective pressure to retain functional genetic variations required for resistance to pathogens and adaptive responses to changing environments.

## Results

### Draft Genome Assemblies of the Kochi and Miyazaki Individuals of Akame

*De novo* assembly of the akame genome (Kochi individual) was generated by “hybrid” genome assembly approach that combined data from Chromium-linked Illumina reads and Nanopore long reads. In the Kochi individuals, 134.3 Gb raw reads were generated by sequencing the Chromium linked-read library using Illumina HiSeq X Ten, and 33.44 Gb raw reads were generated using Nanopore PromethION (number of reads: 3,708,639 and mean read length: 12.78 kb). The haploid genome size was estimated to be 632.9 Mb by the k-mer frequency analysis under the best k-mer length of 91 (supplementary fig. S1, Supplementary Material online). Assembly statistics of the Kochi akame genome are summarized in Table 1. Our hybrid assembly approach generated a genome sequence with high contiguity for akame (10,039 scaffolds, scaffold N50: 3.71 Mb, and assembly size: 837.9 Mb). The genome effective length (=total genome length − number of *N* sites) of the scaffold-level assembly was 790.1 Mb, which was substantially larger than the genome size estimated by k-mer frequency analysis. BUSCO completeness score (=percentage of the presence of core genes in the genome assembly) for the scaffold-level assembly was 93.4% (89.5% single and 3.9% duplicated copies). Contamination of the sequences derived from the microbiome or other organisms in the scaffold-level assembly was virtually undetected by GC content and BLASTX-based genome screening (supplementary fig. S2, Supplementary Material online).

**Table 1:** Statistics of the akame reference genome assembly (Kochi individual)

Statistics	Primary genome assembly (contig)	Primary genome assembly (scaffold)	Scaffold-level assembly (scaffolding with Nanopore long reads and RNA-Seq data)
Number of contigs/scaffolds	55,863	38,165	10,039
Contig/scaffold N50	26,246	1,090,786	3,710,658
Contig/scaffold L50	5,824	172	52
Largest contig/scaffold size	297,685	6,847,197	18,195,970
Total size of the assembly	598,661,182	706,963,282	837,949,969

“Primary genome assembly” indicates the assembly generated using only chromium-linked illumina reads. More detailed statistics of the genome assemblies are summarized in supplementary table S1, Supplementary Material online.

Chromosome-level assembly was also generated by ordering the scaffolds along the *L. calcarifer* genome assembly. Chromosome-level assembly comprised 24 chromosomes (the mitochondrial genome was not included), and the total assembly size was 778.6 Mb. BUSCO completeness score for the chromosome-level assembly was 91.9% (90.3% single and 1.6% duplicated copies). In the remainder of this paper, the scaffold- and chromosome-level assemblies of the Kochi individual are termed “akame (Kochi) genome” and used as references for subsequent analyses. Assembly statistics of the akame (Kochi) genome at each analysis step are shown in supplementary table S1, Supplementary Material online.

We obtained the de novo assembly for the Miyazaki individual of akame by Chromium-linked Illumina reads. In the Miyazaki individual, 125.3 Gb of raw reads were generated by sequencing the Chromium-linked read library using Illumina HiSeq X Ten. The genome of the Miyazaki individual was assembled into 17,019 scaffolds totaling 657.1 Mb in size. The N50 of the assembly was 1,526,648 bp. BUSCO completeness score for the Miyazaki akame assembly was estimated as 95.8% (91.6% single and 4.2% duplicated copies). Similar to the akame (Kochi) genome, sequences derived from potential contaminant organisms were almost negligible in the Miyazaki akame genome assembly (supplementary fig. S3, Supplementary Material online). For the Miyazaki akame individual, details of assembly statistics are shown in supplementary table S2, Supplementary Material online. In the remainder of this paper, this assembly has been termed “akame (Miyazaki) genome.”

### Genome Annotation

In the scaffold-level assembly of the akame (Kochi) genome, we predicted 48,291 protein-coding genes based on transcriptome evidence. DIAMOND-BLASTP searches revealed that 61% of the predicted genes (29,610 genes) had homologs in the NCBI *L. calcarifer* and RefSeq databases (vertebrates: Excluding mammals). This low percentage may be attributed to the fact that the akame gene set contains many short sequences of <300 bp. These were likely partial gene sequences caused by incompleteness of the genome assembly and/or insufficient transcriptome evidence in functional annotation. After the short sequences were excluded, 36,907 predicted genes remained, of which 74% of them (27,175 genes) possessed homologs in the databases. BLASTN searches against the ENSEMBL barramundi transcriptomes showed that 56% (27,333/48,291) of the akame genes had corresponding barramundi homologs. Similarly, the proportion increased to 69% (25,581/36,907) after excluding sequences of <300 bp from the gene set. BUSCO completeness of the annotated protein-coding gene set was 79.1% (75.5% single and 3.6% duplicated copies).

In repeat sequences, transposable elements (TEs) comprised ∼14% (118 Mb) of the akame (Kochi) genome, with DNA transposons being the most abundant (4.12%, 34.5 Mb). Comparison of repeat contents between the akame (Kochi) and barramundi genomes (Vij et al. 2016), which were estimated by the same method (see Materials and Methods), revealed no clear differences. The numbers of TEs, satellites, and simple repeats in the genomes of the two species are shown in supplementary table S3, Supplementary Material online.

### Demographic History of Akame

**Fig. 2.**
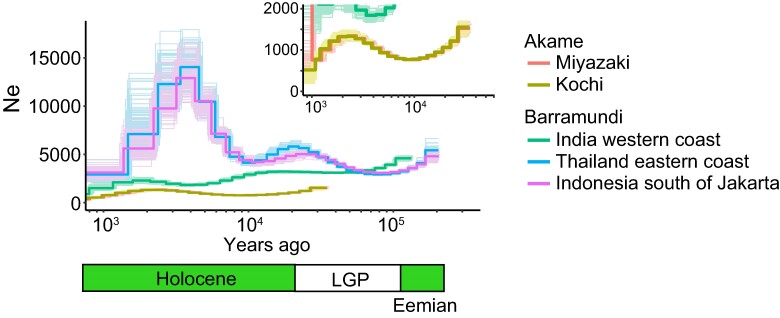
Historical *N*_e_s inferred by PSMC analysis for akame (Kochi and Miyazaki) and barramundi (India, Thailand, and Indonesia). Fine lines denote bootstrap replicates. The green and white bars below indicate the last three interglacial and glacial periods, respectively. LGP, last glacial period.

The inferred *N*_e_ history by pairwise sequentially Markov coalescent (PSMC) analysis revealed that the *N*_e_ of the two akame populations has been maintained around 1,000 from 30,000 years ago to the recent past (Fig. 2), indicating their long-time persistence despite a small population size. By contrast, the *N*_e_ of barramundi populations in Thailand and Indonesia were estimated to be 3 to 10 times larger than that of akame, indicating that the population size expansion and decline had occurred 10^3^ to 10^4^ years ago (Fig. 2). It is interesting to note that the *N*_e_ of the West India barramundi population was constantly low and the population size expansion in the Holocene was not observed (Fig. 2).

### Heterozygosity Levels, Genetic Load, and Runs of Homozygosity of the Akame Genome

The numbers of single nucleotide variant (SNV) sites in the two individuals of akame were counted by mapping the genomic reads to the chromosome-level assembly. In the Kochi individual, 251,220 heterozygous single nucleotide variants (SNVs) were identified and genome-wide heterozygosity was estimated as 3.4 × 10^−4^/bp. In the Miyazaki individual, 245,311 heterozygous SNVs were identified, and genome-wide heterozygosity was 3.3 × 10^−4^/bp, suggesting that the proportions of SNVs were not different between the Kochi and Miyazaki populations. In the barramundi individual from Singapore (SG), 1,100,370 heterozygous SNVs were identified and genome-wide heterozygosity was 16.0 × 10^−4^/bp. Heterozygosity in the akame genomes was not >20% of the SG individual of barramundi.

**Fig. 3.**
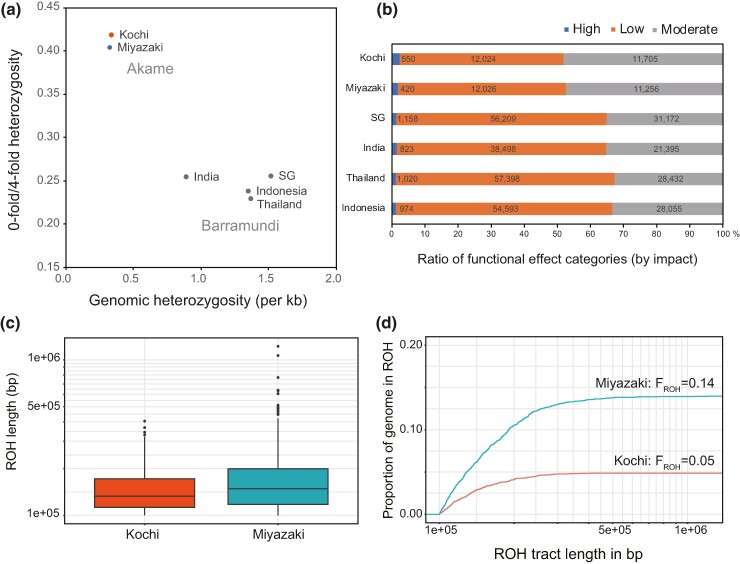
a) A relationship between genomic heterozygosity and ratio of heterozygosity at 0-fold relative to 4-fold degenerate sites in the individuals of akame and barramundi. b) Ratios of functional effect categories of SNVs in the individuals of akame and barramundi. Functional effect categories in each SNV were identified using the SnpEff program (Cingolani et al. 2012). Functional effect categories: High, nonsense and splicing donor/acceptor site variants; Moderate, missense variants; Low, synonymous and stop retained variants. c) Boxplot of the ROH lengths in Kochi and Miyazaki akame individuals. d) Cumulative fraction of the genome made up of ROHs at least 100 kb long.

To assess the genetic load in akame, we estimated the relationship of the genomic heterozygosity (a proxy for *N*_e_) and the ratio of heterozygosity in 0-fold relative to 4-fold degenerate sites within protein-coding regions (a proxy for the efficacy of selection) in akame and compared these to those in barramundi. Genomic heterozygosity was much lower and the ratio of 0-fold/4-fold heterozygosity was much higher in akame than in barramundi (Fig. 3a). Also, the proportions of heterozygous SNVs with “High” and “Moderate” functional effects (see Materials and Methods for detail) were higher in the akame genomes than in the barramundi genomes (Fig. 3b). Similarly, the SIFT missense predictions showed that the ratio of “deleterious” and “tolerated” variants was higher in akame than in barramundi (supplementary fig. S4, Supplementary Material online). The increased proportions of putatively deleterious mutations and the reduction in neutral heterozygosity implied the genomic consequences of enhanced drift relative to selection by long-term small *N*_e_ in akame.

The numbers of runs of homozygosity (ROH) >100 kb were 251 in the Kochi and 616 in Miyazaki individuals. Mean length of the ROH (>100 kb) was 150.8 kb in the Kochi individual and 176.8 kb in the Miyazaki individual (Fig. 3c). In the akame genome, almost all ROHs were <1 Mb in length. Two ROHs > 1 Mb were identified in the Miyazaki individual and not found in the Kochi individual (Fig. 3c). The *F*_ROH_ of the Kochi and Miyazaki individuals was estimated as 0.05 and 0.14, respectively (Fig. 3d). In barramundi (SG individual), ROHs > 100 kb were not found.

### Genome-Wide Distribution of Genetic Variations in the Akame Genomes

**Fig. 4.**
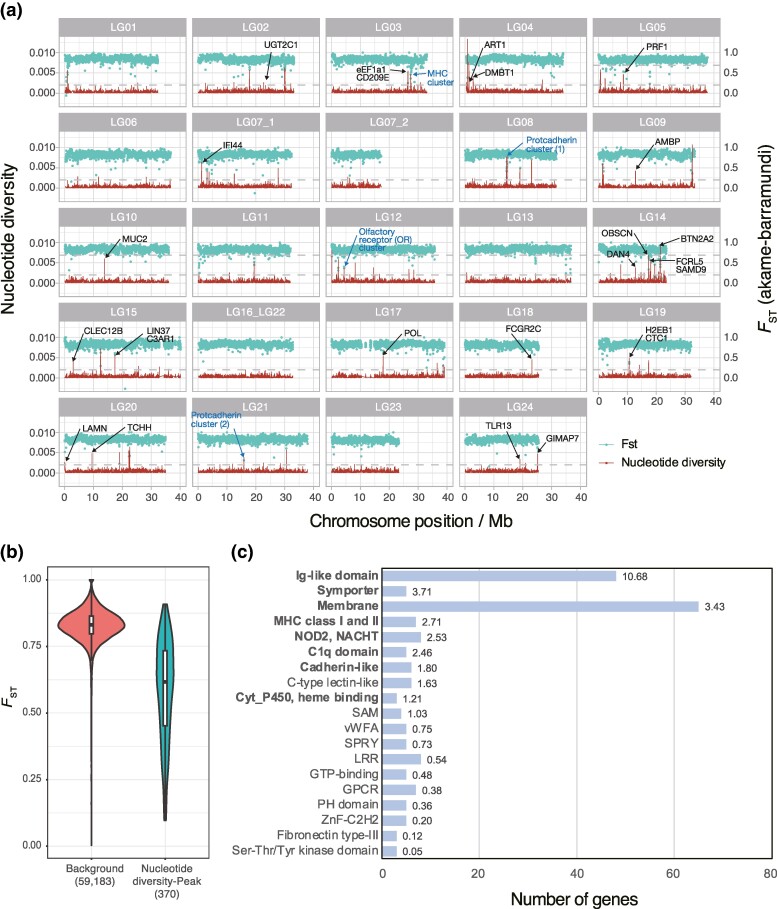
a) A sliding-window plot of nucleotide diversity (red: left *y* axis) and mean *F*_ST_ (light blue: right *y* axis) for 10-kb nonoverlapping windows in the akame genome. The mitochondrial genome was excluded from the analysis. Dashed line indicates the cutoff value of nucleotide diversity (mean + 4 SD: 0.0020). Abbreviations of the representative polymorphic genes in the nucleotide diversity peak regions (nucleotide diversity within the coding region >0.01) are shown in the figure (see Table 2 for details). In addition, the genomic locations of the MHC, OR, and two protocadherin gene clusters within the nucleotide diversity-peak regions are indicated by blue arrows. b) A violin plot of *F*_ST_ values in the background and nucleotide diversity-peak regions. Distribution of *F*_ST_ values in the nucleotide diversity-peak regions was significantly shifted toward lower values compared with background regions (*P* < 2.2 × 10^−16^, two-tailed Mann–Whitney test). Numbers of windows in each category are shown in parentheses. c) Summary of the functional annotation clustering of the 221 genes within the nucleotide diversity-peak regions. The number of genes in each cluster is shown in the graph. Numbers on the right of the bars indicate fold enrichment. The names of significantly enriched (FDR-adjusted *P*-value < 0.05) clusters are indicated in bold. A full list of the annotation clusters is provided in supplementary table S6, Supplementary Material online.

After stringent window filtration by mean mappability and depth of coverage (supplementary figs. S5 and S6, Supplementary Material online; see Materials and Methods), multiple peaks of nucleotide diversity that exceeded the mean + four standard deviation (4 SD) were identified. These peaks were scattered across the multiple genomic regions (Fig. 4a). The number of windows of nucleotide diversity-peaks was 231, and 271 protein-coding genes were detected within them (listed in supplementary table S4, Supplementary Material online).

In many cases, balancing selection can reduce population differentiation (measured by *F*_ST_) around the selected genes or genomic regions (Brandt et al. 2018). If the high polymorphisms in the nucleotide diversity-peak regions in akame are maintained by balancing selection, thus, these regions are likely less divergent to the barramundi genome than the background. To test this, mean *F*_ST_ values between akame (Kochi and Miyazaki) and barramundi (SG, India, Thailand, and Indonesia) were estimated across the 10 kb nonoverlapping window in the chromosome-level assembly. *F*_ST_ values in the nucleotide diversity-peak regions appear to much lower than those in the background regions (Fig. 4a). Indeed, the distribution of *F*_ST_s in the nucleotide diversity-peak regions (median *F*_ST_ = 0.632) was significantly shifted toward lower values, compared to the background regions (median *F*_ST_ = 0.831) (Fig. 4b). To examine whether the nucleotide diversity-peak regions in akame were common in barramundi, nucleotide diversity per 10 kb nonoverlapping window was also calculated using the four barramundi individuals, and chromosomal positions of the nucleotide diversity-peaks were compared between the two species (supplementary fig. S7, Supplementary Material online). In barramundi, 131 windows of nucleotide diversity-peaks were identified. Of these, 44 were common to akame, and 58 genes were contained in these windows. The number of windows with high nucleotide diversity common to the two species was significantly larger than that of randomly expected (*P* < 1 × 10^−6^, Monte Carlo simulation; see Materials and Methods for detail).

Enrichment analysis of the 221 genes within the nucleotide diversity-peak regions that had corresponding ENSEMBL barramundi transcripts (>90% nucleotide sequence identity) showed significant enrichment of the GO terms “translation elongation factor activity” (*P* = 0.0287, GO:0003746), “Cell adhesion” (*P* = 0.0417, GO:0007155), “Membrane” (*P* = 6.0 × 10^−4^, GO:0016020), “MHC protein complex” (*P* = 0.0137, GO:0042611), and “MHC Class II protein complex” (*P* = 0.0137, GO:0042613). Detailed results of the enrichment analysis are shown in supplementary table S5, Supplementary Material online. In 98 of the 271 genes located within the nucleotide diversity-peak regions, the proportion of SNV sites within the protein-coding region was >0.01. In this paper, we refer to these genes as “polymorphic genes.” The top 30 of the polymorphic genes are listed in Table 2.

**Table 2:** A list of the top 30 polymorphic genes located within the nucleotide diversity-peak regions

Linkage group	Start	End	Ori.	Length of_CDS	Transcript ID (ENSEMBL barramundi)	Number of variants (LOW)	Number of variants (MODERATE)	Number of variants (HIGH)	Proportion of variants within CDS	Gene name (abbreviation)	Description (BLASTP-DIAMOND best hit)
LG03	26449453	26465165	−	456	ENSLCAT00010049262	8	29	3	0.0877	*CD209E*	CD209 antigen-like protein E isoform X1 [*L. calcarifer*]
LG14	12799161	12800000	−	309	…	16	9	0	0.0809	*DAN4*	Cell wall protein DAN4-like isoform X2 [*L. calcarifer*]
LG24	25153065	25153736	+	672	ENSLCAT00010055642	8	41	0	0.0729	*GIMAP7*	GTPase IMAP family member 7-like [*L. calcarifer*]
LG15	2952166	2954872	+	885	ENSLCAT00010019791	8	53	1	0.0701	*CLEC12B*	C-type lectin domain family 12 member B-like isoform X3 [*L. calcarifer*]
LG02	22741864	22742424	−	561	ENSLCAT00010041310	18	18	0	0.0642	*UGT2C1*	UDP-glucuronosyltransferase 2C1-like isoform X2 [*L. calcarifer*]
LG12	4346931	4348396	+	474	ENSLCAT00010002461	9	17	0	0.0549	*OR4K1*	Olfactory receptor 4K1-like [*L. calcarifer*]
LG14	18061006	18063156	−	2151	…	25	92	0	0.0544	*SAMD9*	Sterile alpha motif domain-containing protein 9-like [*Epinephelus lanceolatu*s]
LG03	26294625	26296711	+	456	ENSLCAT00010027654	9	14	0	0.0504	*eEF1a1*	Elongation factor 1-alpha-like [*L. calcarifer*]
LG07_1	1237419	1240000	−	666	ENSLCAT00010040075	6	27	0	0.0495	*IFI44*	Interferon-induced protein 44-like [*L. calcarifer*]
LG20	9471714	9473135	+	1227	…	21	38	1	0.0489	*TCHH*	Trichohyalin-like [*L. calcarifer*]
LG03	26466013	26468750	−	309	ENSLCAT00010011964	1	13	1	0.0485	*CD209E*	CD209 antigen-like protein E Isoform X2 [*L. calcarifer*]
LG15	17396770	17397786	−	1017	ENSLCAT00010017757	15	31	0	0.0452	*C3AR1*	C3a anaphylatoxin chemotactic receptor-like [*L. calcarifer*]
LG04	2823523	2833145	+	954	ENSLCAT00010002680	14	25	1	0.0419	*DMBT1*	Deleted in malignant brain tumors 1 protein-like [*L. calcarifer*]
LG14	17330002	17334406	+	1986	ENSLCAT00010036890	21	50	2	0.0368	*OBSCN*	Obscurin-like [*L. calcarifer*]
LG05	8597156	8600000	+	1293	ENSLCAT00010025942	10	28	1	0.0302	*PRF1*	Perforin-1-like [*L. calcarifer*]
LG04	562611	570777	+	834	ENSLCAT00010010642	2	23	0	0.0300	*ART1*	T-cell ecto-ADP-ribosyltransferase 1-like isoform X1 [*L. calcarifer*]
LG10	13782127	13786224	+	2142	ENSLCAT00010023093	26	36	0	0.0289	*MUC2*	Mucin-2-like isoform X1 [*L. calcarifer*]
LG14	17380697	17383457	−	834	ENSLCAT00010036890	9	15	0	0.0288	*FCRL5*	Fc receptor-like protein 5 [*L. calcarifer*]
LG20	236485	240000	−	1557	ENSLCAT00010038704	4	38	1	0.0276	*LAMN*	Lamin-L(II)-like [*L. calcarifer*]
LG19	10632514	10633673	−	297	ENSLCAT00010060760	3	5	0	0.0269	*H2EB1*	H-2 Class II histocompatibility antigen, E-S beta chain-like [*L. calcarifer*]
LG12	4352144	4359193	+	747	ENSLCAT00010002441	10	10	0	0.0268	*OR52K1*	Olfactory receptor 52K1-like [*L. calcarifer*]
LG17	17846158	17849546	+	936	ENSLCAT00010006135	4	18	3	0.0267	*POL*	RNA-directed DNA polymerase from mobile element jockey-like [*Austrofundulus limnaeus*]
LG15	17390002	17391523	+	618	ENSLCAT00010017781	6	7	1	0.0227	*LIN37*	Protein lin-37 homolog isoform X2 [*L. calcarifer*]
LG14	21371009	21373848	−	810	ENSLCAT00010060848	8	10	0	0.0222	*BTN2A2*	Butyrophilin subfamily 2 member A2-like isoform X1 [*L. calcarifer*]
LG09	12822294	12823545	+	273	ENSLCAT00010036814	2	3	1	0.0220	*AMBP*	Protein AMBP-like [*L. calcarifer*]
LG24	19030002	19032243	−	2886	ENSLCAT00010001000	24	38	1	0.0218	*TLR13*	Toll-like receptor 13 [*L. calcarifer*]
LG19	10650959	10651986	−	507	…	6	5	0	0.0217	*CTC1*	CST complex subunit CTC1-like [*L. calcarifer*]
LG03	26291502	26292790	+	753	ENSLCAT00010027940	7	9	0	0.0212	*eEF1a1*	Elongation factor 1-alpha-like [*L. calcarifer*]
LG18	23174362	23177107	−	714	…	6	9	0	0.0210	*FCGR2C*	Low affinity immunoglobulin gamma Fc region receptor II-a-like isoform X2 [*L. calcarifer*]
LG03	26296792	26297830	+	336	ENSLCAT00010028429	1	6	0	0.0208	*eEF1a1*	Elongation factor 1-alpha [*L. calcarifer*]

Genes described as “uncharacterized protein” are not included in this table. A full list of the genes within the nucleotide diversity-peak regions (272 genes) is shown in supplementary table S4, Supplementary Material online. The numbers of SNVs are shown by annotation impacts (by SnpEff) as follows: LOW, synonymous or stop retained variants; MODERATE, missense variants; HIGH, nonsense, start lost, or splicing donor/acceptor variants.

Functional annotation clustering using the DAVID web tools identified 19 clusters from the 221 genes (Fig. 4c). Similar to the GO enrichment analysis, the DAVID analysis also suggested that the genes involved in innate and adaptive immune systems (Ig-like domain, MHC Class I and II, C1q domain, C-type lectin-like, etc.) and cell adhesion (Cadherin-like) were significantly enriched in the nucleotide diversity-peak regions (see supplementary table S6, Supplementary Material online).

### Selective Pressure and Evolutionary Rates in the Akame Genes

Conducting the ortholog-detection method by Yang and Smith (2014) (see Materials and Methods for detail), we identified 8,825 gene sets consisting of 6,847 one-to-one and 1,978 maximum inclusion (MI) orthologs in the five species examined (akame, barramundi, perch, medaka, and seahorse). A robust phylogenetic relationship of the five fishes was reconstructed using all one-to-one ortholog gene set (supplementary fig. S8, Supplementary Material online), to apply the following evolutionary analyses.

**Fig. 5.**
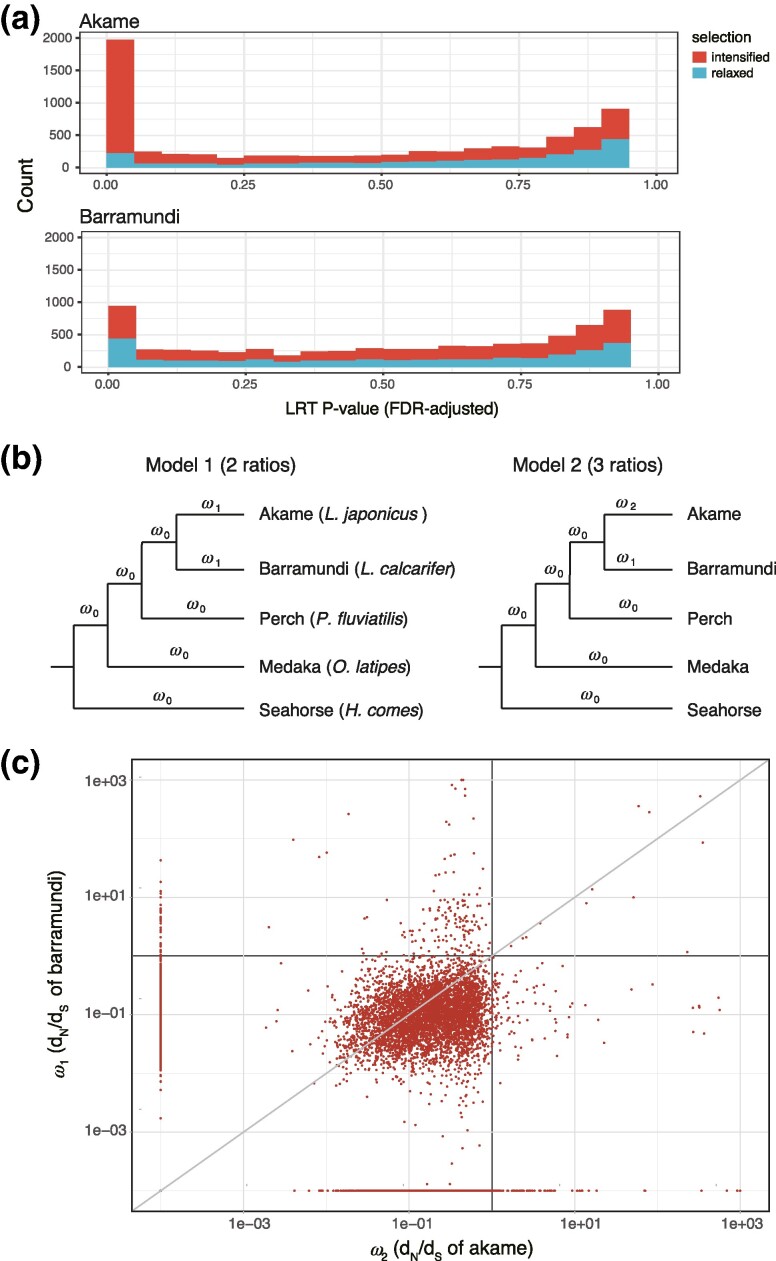
Analysis of selection in akame genes. a) FDR-adjusted *P*-value distributions of the RELAX tests of akame (above) and barramundi (below). Red and blue bars indicate the numbers of genes with intensified (*k* > 1) and relaxed (*k* < 1) selective pressures, respectively. For visualization, the numbers of genes with a *P*-value of >0.95 were excluded from the histograms. b) Two branch-specific models to test differences in selective pressures between akame and barramundi. Phylogenetic relationship of the five fish species was reconstructed by the maximum-likelihood methods using concatenated one-to-one orthologous gene set (supplementary fig. S8, Supplementary Material online; see Materials and Methods). c) Scatter plot of the synonymous–nonsynonymous nucleotide substitution rate ratio (*ω* = *d*_N_/*d*_S_) in the branches of akame (*ω*_2_, *x* axis) and barramundi (*ω*_1_, *y* axis) estimated by Model 2 in each orthologous gene set. For visualization, genes with *ω*_1_ and/or *ω*_2_ values estimated as infinity were excluded from the plot. Genes with *ω*_1_ and/or *ω*_2_ estimated as 0 were included in the plot by adjusting them to replace 1 × 10^−4^. Diagonal line indicates *ω*_1_ = *ω*_2_.

The level of the *N*_e_ determines the effectiveness of selection relative to drift (Charlesworth 2009). Thus, a reduction in *N*_e_ indicates the efficacy of selection and can cause fixations of mildly deleterious mutations. To investigate whether the relaxation of selective pressure has occurred in akame after speciation to barramundi, a selection intensity in akame and barramundi was inferred using the RELAX descriptive models (Wertheim et al. 2015) in all one-to-one and MI ortholog gene sets (see Materials and Methods for details of this analysis). *P*-value distributions of the RELAX tests for akame and barramundi are shown in Fig. 5a. In akame, contrary to our expectation, the number of genes under intensified selective pressures (1,754 genes, FDR-adjusted *P* < 0.05) was much larger than that under relaxation of selection (223 genes, FDR-adjusted *P* < 0.05). By contrast, in barramundi, the number of genes under intensified selective pressures (462 genes, FDR-adjusted *P* < 0.05) was comparable to that under relaxed selective pressures (417 genes, FDR-adjusted *P* < 0.05). A scatter plot of *k* in akame (Kochi) versus barramundi also indicated that the number of genes under intensified selective pressures was much larger in akame than in barramundi (supplementary fig. S9, Supplementary Material online).

We tested two branch-specific models to examine the evolutionary rate differences between akame and barramundi (Fig. 5b). In Model 1 (two-ratio), two *ω* values are defined in the branches connecting to akame and barramundi (*ω*_1_) and in other “background” branches (*ω*_0_). In Model 2 (three-ratio), three *ω* values are defined, in the branch connecting to akame (*ω*_2_), in the branch connecting to barramundi (*ω*_1_), and in other background branches (*ω*_0_). In each of the orthologous gene set, parameters and log-likelihood values were estimated, and the validity of Model 2 was tested by the likelihood ratio test (LRT). The branch-specific tests showed that selective pressure was significantly different between akame and barramundi in 238 genes (FDR-adjusted *P*-value < 0.05). In most of these genes (222 genes), *ω* values in the branch connecting to akame were larger than those in the branch connecting to barramundi (*ω*_2_ > *ω*_1_), and 131 and 2 of these genes were suggested to have evolved under intensified and relaxed selective pressures by RELAX analysis, respectively (*k* < 1 and FDR-adjusted *P* < 0.05; see supplementary table S8-(a), Supplementary Material online). On the other hand, genes that *ω* values in the barramundi branch were greater than those in the akame branch were only 16 (*ω*_2_ <*ω*_1_), and 4 and 1 of them were suggested to have evolved under intensified and relaxed selective pressures, respectively (*k* < 1 and FDR-adjusted *P* < 0.05; see supplementary table S9-(a), Supplementary Material online). The analysis also suggested that the *ω*_2_ values exceeded 1 in the 15 akame-specific fast-evolving genes, and 7 of them were suggested to have evolved under intensified selective pressure (supplementary table S10, Supplementary Material online). Distribution of *ω*_1_ (barramundi) versus *ω*_2_ (akame) of Model 2 in every orthologous gene pair clearly showed that a relatively larger number of genes were plotted below the diagonal, implying an elevated evolutionary rate in akame (Fig. 5c). The number of genes that showed higher *ω* values in akame was significantly greater than that of barramundi for all genes analyzed (*P* < 2.2 × 10^−16^, exact binomial test) and in the genes that Model 2 was supported statistically (*P* < 2.2 × 10^−16^, exact binomial test). GO enrichment analysis of genes with higher *ω* values in akame (222 genes: Model 2 was supported by LRT) showed that the terms “calcium ion transmembrane transporter activity” (*P* = 3.2 × 10^−4^, GO:0015085), “divalent inorganic cation transmembrane transporter activity” (*P* = 3.5 × 10^−4^, GO:0072509), “calcium channel activity” (*P* = 3.4 × 10^−3^, GO:0005262), “metal ion transmembrane transporter activity” (*P* = 0.037, GO:0046873), “calcium ion transmembrane transport” (*P* = 2.0 × 10^−4^, GO:0070588), “calcium ion transport” (*P* = 2.6 × 10^−3^, GO:0006816), “divalent metal ion transport” (*P* = 8.3 × 10^−3^, GO:0070838), “divalent inorganic cation transport” (*P* = 8.8 × 10^−3^, GO:0072511), and “regulation of localization” (*P* = 0.025, GO:0032879) were significantly enriched in the akame-specific rapidly evolving genes. By contrast, the terms “eye-pigmentation genes” (*P* = 1.1 × 10^−3^, GO:0048069) and “vacuolar proton-transporting V-type ATPase, V1 domain” (*P* = 0.050, GO:0000221) were enriched in the barramundi-specific rapidly evolving genes (16 genes). Detailed results of the enrichment analysis are described in supplementary tables S8-(b) and S9-(b), Supplementary Material online.

## Discussion

### Extremely Low Level of Heterozygosity in the Akame Genomes

In this study, the genome-wide single nucleotide heterozygosity levels of the Kochi and Miyazaki akame genomes were estimated to be 3.4 × 10^−4^/bp and 3.3 × 10^−4^/bp, respectively. These were consistent with the genetic diversity estimates based on AFLP (Takahashi et al. 2015) and ddRAD-Seq (Naito et al. 2023). The level of heterozygosity in akame is comparable to that in some critically endangered vertebrate species (Table 3). The heterozygosity of akame is substantially lower than those of brackish (three-spined stickleback) and marine (yellow croaker) fish species, slightly lower than that of sunfish *Mola mola*, and similar to that of the long-lived Pacific Ocean rockfish *Sebastes levis*, which exhibits the lowest genomic heterozygosity in teleost fishes (Kolora et al. 2021). Thus, akame is considered to be one of the marine teleost fishes with the lowest genetic diversity. The level of heterozygosity in akame is <20% of that of the barramundi (SG individual; see Results). The unusually low heterozygosity in akame is likely caused by the long-term suppression of *N*_e_ in this species. Estimation of historical population size using the PSMC model suggested that the small *N*_e_ of akame (*N*_e_ = 500 to 1,500) has been sustained from ∼3 × 10^4^ years ago to the recent past (Fig. 2). By contrast, the *N*_e_s of the Thailand and Indonesia populations of barramundi have been maintained at an order of 10^3^ from the last glacial period (LGP) to Holocene, and they have experienced a rapid population expansion, following a bottleneck in 10^3^ to 10^4^ years ago (Fig. 2).

**Table 3:** Levels of genome-wide SNP heterozygosity in akame, barramundi, four marine teleost fishes, and six endangered birds and mammals

Common name	Scientific name	Group	SNP heterozygosity of whole genome (10^−4^ /bp)	IUCN red list category^a^	References
Akame (Kochi)	*L. japonicus*	Teleost fish	3.4	VU	This study
Akame (Miyazaki)	*L. japonicus*	Teleost fish	3.3	VU	This study
Barramundi (SG, Singapore)	*L. calcarifer*	Teleost fish	19.0	LC	Estimated from the data presented in Vij et al. (2016)
Three-spined stickleback	*G. aculeatus*	Teleost fish	14.3	LC	Jones et al. (2012)
Yellow croaker	*Larimichthys crocea*	Teleost fish	35.8	CR	Wu et al. (2014)
Ocean sunfish	*M. mola*	Teleost fish	7.8	VU	Pan et al. (2016)
Ocean rockfish	*S. levis*	Teleost fish	2.9	…	Kolora et al. (2021)
Crested ibis	*N. nippon*	Bird	4.3	EN	Li et al. (2014)
White-tailed eagle	*Haliaeetus albicilla*	Bird	4	LC	Li et al. (2014)
Bald eagle	*Haliaeetus leucocephalus*	Bird	4.3	LC	Li et al. (2014)
Island fox	*U. littoralis*	Mammal	0.142 to 4.08	NT	Robinson et al. (2016)
Mountain gorilla	*G. gorilla*	Mammal	6.5	EN	Xue et al. (2015)
Greater bamboo lemur	*P. simus*	Mammal	0.37	CR	Hawkins et al. (2018)

^a^IUCN red list categories: CR, critically endangered; EN, endangered; VU, vulnerable; NT, near endangered; and LC, least concern.

The PSMC analysis also suggested that the historical *N*_e_ of akame was much smaller than those reported for other critically endangered vertebrate species. In crested ibis *Nipponia nippon*, *N*_e_ had been maintained at 2 to 6 × 10^4^ from 1 MYA to the LGP and then fell to ∼ 1 × 10^4^ at the end of the LGP. One wild population of crested ibis declined to only seven individuals from two breeding pairs in the middle of the 20th century (Li et al. 2014). The *N*_e_ of the mainland population of gray fox *U. littoralis* had been kept ∼ 1 × 10^4^ from 10^5^ to 10^3^ years ago and decreased to ∼ 2 × 10^3^ at 100 years ago (Robinson et al. 2016). Historical *N*_e_ of greater bamboo lemur *Prolemur simus* was estimated between 9 × 10^5^ to 1 million individuals in 6 to 9 × 10^4^ years ago and declined to 1 to 4 × 10^3^ at present (Hawkins et al. 2018). It is important to note that these endangered species commonly experienced severe population decline during the LGP and the recent past (19th to 20th centuries). By contrast, the *N*_e_ of akame was small but stable for long periods and does not appear to have undergone a rapid decline during the LGP, although the recent population size changes (from the 19th to 20th century to the present) in this species are unknown. The dynamics of the historical population size in akame may be more similar to that of the mountain gorilla *G. beringei beringei*, which is thought to have maintained a small population size (*N*_e_ < 10^3^) from 10^5^ years ago to the recent past (Xue et al. 2015). It is revealed that the population of the mountain gorilla has remained relatively stable (Xue et al. 2015; van der Valk et al. 2019). In the mountain gorilla, a decrease in genetic diversity and an increase in genetic load have not been observed in recent centuries (van der Valk et al. 2019), which can be attributed to the purging of deleterious recessive alleles as a result of the constant small population size in this subspecies. On the other hand, the genetic load was suggested to be substantially higher in akame than in barramundi (Fig. 3a and b, and supplementary fig. S4, Supplementary Material online). Also, ROH length and *F*_ROH_ were relatively smaller in akame (Fig. 3c and d) than in species experiencing severe inbreeding (e.g. Xie et al. 2022). Taken together, in contrast to the case of the mountain gorilla, purging deleterious alleles is unlikely to occur in akame, despite the long-term small *N*_e_ in this species.

It must be emphasized that the relatively stable historical *N*_e_ in akame does not necessarily mean its robustness to rapid population size decline due to human activities. Recently, there has been increasing concern over the akame population decline due to strong fishing pressure and loss of eelgrass (*Zostera japonica*) bed habitats, which are essential for the larvae and juveniles of this species (Kinoshita et al. 1988). Population size changes in akame in recent centuries are not well understood because census population size has not been adequately estimated. Close-kin mark–recapture (CKMR) analysis is a new genetic marker-based method for assessing population abundance, by estimating census population size directly from population genomic data (Bravington et al. 2016). Recent studies have successfully applied CKMR to estimate population abundances in marine and freshwater fishes (Bravington et al. 2016; Ruzzante et al. 2019). Our results will provide basic genomic information for the application of CKMR in akame.

### Genes in the Nucleotide Diversity-Peak Regions of the Akame Genomes

The sliding-window analysis revealed extremely low nucleotide diversity in most chromosomal regions in the akame genome (Fig. 4a). However, the analysis identified multiple genomic regions with significantly high nucleotide diversity, within which 271 genes were identified (supplementary table S4, Supplementary Material online). Analysis of window-*F*_ST_ between akame and barramundi genomes showed that the nucleotide diversity-peak regions were significantly less divergent than the background regions (Fig. 4b), suggesting that the polymorphisms in akame genome were retained by balancing selection. Enrichment analysis of the genes within the nucleotide diversity-peak regions indicated that genes involved in the immune systems were significantly enriched (Fig. 4c; supplementary table S5, Supplementary Material online). In many organisms including fishes, polymorphisms of immune genes are retained by balancing selection in order to keep resistance against diverse pathogens (Croze et al. 2016; Yamaguchi and Dijkstra 2019). For example, a gene cluster of the major histocompatibility complex (MHC) is a prime example of immune genes of which polymorphisms were retained by balancing selection (Spurgin and Richardson 2010), was found in the nucleotide diversity-peak region in LG03 (Fig. 4a). One nonsense variant was identified in CD209 antigen-like protein E (*CD209E*) gene in LG03 (Fig. 4a). *CD209E* is one of the homologs of C-type lectin 4 (CTL4) proteins that are used for recognizing pathogens in the innate immune system of fishes and mammals (Xue et al. 2018). Enrichment analysis also showed that genes involved in cell–cell adhesion are significantly enriched in the nucleotide diversity-peak regions. This is mainly caused by two protocadherin gene clusters located in LG08 and LG21 (Fig. 4a, supplementary table S4, Supplementary Material online). Protocadherins are members of the cadherin superfamily and are associated with synaptic development in the vertebrate brain (Morishita and Yagi 2007). In humans, signatures of balancing selection were detected in the promoter regions of protocadherin α gene clusters (Noonan et al. 2003). In addition, four olfactory receptor (OR) genes and one trace amine-associated receptor (TAAR) gene were included in the nucleotide diversity-peak regions (supplementary table S4, Supplementary Material online). In humans and teleost fishes, the diversity of OR genes is suggested to be maintained by balancing selection (Olender et al. 2012; Liu et al. 2021). In akame, it is likely that a part of the genetic polymorphisms of the genes and/or gene regulatory regions within the nucleotide diversity-peak regions are maintained by balancing selection, despite the low level of genetic diversity. Comparison of the sliding windows of nucleotide diversity between akame and barramundi revealed that 44 windows of the nucleotide diversity-peak regions in akame were common to barramundi (supplementary fig. S7, Supplementary Material online), suggesting that polymorphisms in these peaks were retained from the common ancestor of the two species. Genomic regions containing protocadherin (LG08) and OR (LG12) gene clusters were commonly polymorphic both in akame and barramundi (supplementary fig. S7, Supplementary Material online). Interestingly, akame-specific polymorphic genomic regions were also identified. For example, some of the polymorphic genes involved in immunity, such as *CD209E* in LG03, interferon-induced protein 44-like (*IF44*) in LG07_1, and butyrophilin subfamily 2 member A2-like (*BTN2A2*) in LG14, were located in these akame-specific polymorphic regions (supplementary fig. S7, Supplementary Material online). It seems that high polymorphisms in these regions have occurred after the speciation of akame and barramundi. Thus, both long- and short-term balancing selections likely contribute to the maintenance of functional genetic variations and the long-term persistence of the natural populations of akame. Our results also emphasize the importance of functional genetic variation for the conservation of endangered species (Teixeira and Huber 2021).

### Selective Pressures for the Akame Genes

The RELAX analysis showed that the proportion of genes under intensified selective pressures was much larger in akame than in barramundi (Fig. 5a and supplementary fig. S9, Supplementary Material online), suggesting that selective pressures have intensified on many akame genes following the speciation of akame and barramundi. This is an unexpected result because the extremely small *N*_e_ of akame likely weakens purifying selection and could lead to the fixation of deleterious mutations in the akame genome. The branch-specific tests indicated that *ω* values tended to be larger in akame genes than in barramundi genes (Fig. 5c). Many of the genes that show higher *ω* values in akame supported by the branch-specific test (131/222 genes) were assigned to the genes under intensified selective pressure by the RELAX analysis, whereas only two genes were assigned to the genes under relaxed selective pressure. Given the increasing selective pressures on these akame genes, this trend, at least partially, could be explained by positive selection likely caused by some akame-specific rapid adaptations that occurred after the speciation of akame and barramundi. It is important to note that, however, intensified selective pressures in these genes may reflect past adaptive evolution in this species, not ongoing ones. Moreover, it seems very unlikely that most of the akame genes under intensified selective pressure (1,754 genes; Fig. 5a) have evolved under positive selection. At present, it is difficult to provide a comprehensive explanation for the trend of the intensified selective pressure on akame genes, and further studies, such as the population genetics-based approach, are required.

The genus *Lates* contains 11 extant species mainly distributed in tropical waters, 7 of which are restricted to freshwater environments (Nelson et al. 2016). Akame is the only latid species that is distributed in temperate waters (Katayama and Taki 1984; Iwatsuki et al. 1993). Following the speciation of akame and barramundi, akame likely underwent adaptation to estuarine conditions in temperate waters. Many akame genes may have evolved under positive selection during the speciation process. Genes responsible for cold tolerance and thermal sensing emerge as primary candidates for adaptive evolution, given the necessity for akame to adapt to low-temperature conditions in the winter months of temperate waters. GO analysis of the akame-specific fast-evolving genes (supplementary table S8-(b), Supplementary Material online) revealed significant enrichment of the genes involved in cation transport. Multiple voltage-dependent potassium and calcium channels and transient receptor potential melastatin (TRPM) channels were included within them. A potassium channel expressed in the cardiac muscle was shown to be activated in cold-acclimated goldfish, suggesting that potassium channels play important roles in cold tolerance (Ganim et al. 1998). In three-spined stickleback *Gasterosteus aculeatus*, the epithelial Ca^2+^ channel is expressed substantially in the freshwater ecotype, which exhibits higher cold tolerance than its marine counterpart (Gibbons et al. 2016). In addition, TRPM channels are also well-known as cold stimuli and menthol sensors (McKemy et al. 2002; Peier et al. 2002; Himmel et al. 2019). Thus, the observed accelerated evolution of cation channels in akame is plausibly linked to enhancing cold tolerance and/or sensory mechanisms in the context of temperate waters.

The synonymous–nonsynonymous substitution rate ratios in the akame branch (*ω*_2_) exceeded 1 in the 15 akame-specific fast-evolving genes and intensified selective pressure was suggested in 7 of these genes (supplementary table S10, Supplementary Material online). In the akame lineage, the seven genes might be involved in the adaptation of temperate waters. For example, *Fatty acid desaturase 2* (*Fads2*) encodes an enzyme catalyzing desaturation in DHA biosynthesis, and is known to be a key metabolic gene for freshwater colonization of *G. aculeatus* (Ishikawa et al. 2019). *Fads2* is also suggested to be involved in the freshwater adaptation of other euryhaline fishes (Ishikawa et al. 2019, 2022). In akame, the adaptive evolution of *Fads2* might have played a role in the colonization of brackish waters in the southern part of Japan. However, the biological functions of fast-evolving genes in akame are largely unknown, and further studies are needed to understand the adaptive changes that occurred during the speciation of akame and barramundi.

## Conclusion

In this study, we present the draft genome sequences of two individuals of akame. These draft genomes provide essential basic information for future conservation studies of this endangered marine fish species. We show that the akame genomes exhibit extremely low genetic diversity, and the population has persisted over a long period (*ca.* 30,000 years) with small *N*_e_. Similar to the case of mountain gorilla (Xue et al. 2015; van der Valk et al. 2019), the low genetic diversity of akame is likely caused by fixation due to random genetic drift occurring in long-term stable small populations. However, in contrast to the mountain gorilla, purging of deleterious variants was unlikely in akame, because of the elevated levels of genetic load inferred in this species. Analysis of nucleotide diversity-peak regions revealed that high genetic polymorphisms of some immune, synaptic development, and sensory-related genes were maintained within the population, possibly by balancing selection. This implies that akame has a genetic mechanism to maintain functional genetic variation in specific genomic regions that are required for resistance against pathogens and adaptive response to changing environments. This maintenance of functional genetic diversity despite the stable small *N*_e_ possibly contributed to the long-term persistence of this species.

## Materials and Methods

### Sample Collection

For de novo genome sequencing, a blood sample was collected from one juvenile akame individual at the Shimanto river, Kochi Prefecture, Japan (2017 October 20). A blood sample was also collected from one individual at the Shiomi river in Miyazaki Prefecture, Japan (2017 October 4) (Fig. 1). The blood samples were preserved in 99% ethanol until used. In Kochi individual, brain, muscle, and liver samples were also collected from the same individual for transcriptome analysis. In both localities, the wild individuals of akame were collected under special permission from the Divisions of inland water fisheries in Kochi (No. 863) and Miyazaki (No. 21) Prefectures, respectively.

### DNA Extraction, Library Preparation, and Genome Sequencing

Genomic DNA samples were extracted from the red blood cells using a phenol–chloroform method. The quality of genomic DNA was assessed by automated gel electrophoresis using 2200 TapeStation (Agilent Technologies, Santa Clara, CA, USA) and average DNA fragment length was confirmed to be above 60 kb. The concentration of the genomic DNA samples was quantified using microfluorimetry (Qubit Broad range dsDNA Kit, Invitrogen, Carlsbad, CA, USA). From the genomic DNA of the Kochi individual, 10× Genomics Chromium Library was constructed using the Chromium Genome Library Kit and Gel Bead Kit v2, and Chromium Controller and Next Gen Accessory Kit (10× Genomics, Pleasanton, CA, USA) following the manufacturer's protocols. The library was sequenced on a paired-end 2 × 150 nt lane on a HiSeq X Ten (Illumina, San Diego, CA, USA) for a total of 895,181,812 sequence reads. To obtain long DNA reads for scaffolding and gap closing in the assembly, the genome of the Kochi individual was also sequenced by Nanopore PromethION (Oxford Nanopore Technologies, Oxford, UK) following the manufacturer's protocols. For the Miyazaki individual, 10× Genomics Chromium library was also constructed and sequenced on a paired-end 2 × 150 nt lane on a HiSeq X Ten for a total of 835,621,234 sequence reads.

### RNA-Sequencing

To improve genome assembly and gene prediction, transcriptome sequences in akame were obtained by RNA-Seq. Total RNAs of brain, muscle, and liver from the Kochi individual used for genome sequencing were extracted using a PureLink RNA extraction kit (Thermo Fisher Scientific, Waltham, MA, USA) with TRIZOL reagent (Thermo Fisher Scientific) following the manufacturer's protocol. The concentration and RNA integrity number (RIN) of total RNA were measured by Agilent Bioanalyzer 2100. High-quality total RNA (RIN > 7.0; Bioanalyzer 2100 RNA nano kit, Agilent Technologies) was stored at −80 °C until use. Reverse-transcribed cDNA libraries were prepared using TruSeq Stranded mRNA LT Sample Prep Kit (Illumina) and sequenced by paired-end 2 × 100 nt on Illumina platform, following the manufacturer's protocol.

### Genome Size Estimation

To estimate the genome size of akame, a k-mer frequency analysis was performed using KmerGenie (Chikhi and Medvedev 2013). First, in the Kochi individual, 10× barcode sequences of the Chromium linked-reads were trimmed by Long Ranger basic pipeline (10× Genomics). Second, the best k-mer length was estimated from the barcode-trimmed reads. Third, genome size was estimated from the frequency distribution of the best k-mer length.

### Genome Assembly

The procedure used for the hybrid genome assembly approach of the Kochi individual is shown in supplementary fig. S10, Supplementary Material online. First, the Chromium linked-reads of the Kochi individual were assembled using Supernova version 2.1.1 (Weisenfeld et al. 2017) with default settings. Second, the Nanopore long reads were used for scaffolding the linked-reads assembly using SSPACE-LongRead (Boetzer and Pirovano 2014). Third, scaffolding with RNA-Seq reads (see next section) was performed using Rascaf (Song et al. 2016). Gaps in the scaffolds were filled by Nanopore long reads using LR_GapCloser (Xu et al. 2019). Finally, the assembly was polished automatically with the Pilon software tool (Walker et al. 2014) using Illumina linked-reads mapped to the scaffold sequences by BWA (Li and Durbin 2009). Assembly statistics in each analysis step were estimated by assemblathon_stats.pl script (Bradnam et al. 2013). To analyze the chromosomal distribution of polymorphic sites, a chromosome-level assembly was also generated by ordering the scaffolds using mScaffolder (Chakraborty et al. 2018) guided by their alignments to the chromosome-level genome assembly of *L. calcarifer* (Vij et al. 2016) using Nucmer implemented in MUMmer (Kurtz et al. 2004). The Chromium reads of the Miyazaki individual were also assembled by using Supernova version 2.1.1. The quality of the genome assemblies was evaluated by calculating the completeness in BUSCO version 5.1.2 (Simão et al. 2015) with a database of ray-finned fishes (Actinopterygii odb10). To assess the levels of contamination in genome assembly, the proportions of potential contaminants in the akame (Kochi and Miyazaki) genome assemblies were calculated, and visualized by generating BlobPlot, implemented in BlobTools (Laetsch and Blaxter 2017).

### Annotation of Genes and Repeats

Both homology-based and de novo prediction methods were applied to identify repeat sequences in the akame (Kochi) genome assembly. For the homology-based analysis, Repbase (version 20170127) was used to perform searches for TE with RepeatMasker program version 4.1.0 (http://www.repeatmasker.org). For de novo predictions, TEs were identified using RepeatMasker with a de novo repeat library constructed using RepeatModeler version 2.0.1 (Flynn et al. 2020).

Genes in the akame (Kochi) genome (in both scaffold- and chromosome-level assemblies) were predicted using transcript-based methods. First, RNA-Seq reads from the brain, muscle, and liver were cleaned and trimmed by FaQCs (Lo and Chain 2014) and aligned to the soft-masked genome assembly using HISAT2 version 2.1.0 (Kim et al. 2019), and a sorted binary format alignment/map (BAM) file was generated by SAMtools version 1.7 (Li et al. 2009). Second, gene predictions were carried out using the masked genome and RNA-Seq spliced alignment information by GeneMark-ET (Lomsadze et al. 2014) and AUGUSTUS (Keller et al. 2011) implemented in the BRAKER1 pipeline (Hoff et al. 2019). Third, functional information for all putative genes was retrieved by aligning the predicted peptide sequences to the NCBI *L. calcarifer* transcripts (https://www.ncbi.nlm.nih.gov/genome/14180?genome_assembly_id=275271) using DIAMOND-BLASTP searches (*E*-value < 1e−10). Functional information was also retrieved from NCBI RefSeq database (https://www.ncbi.nlm.nih.gov/refseq/) using DIAMOND-BLASTP searches (*E*-value < 1e−10). In addition, to identify corresponding barramundi ENSEMBL transcript IDs, BLASTN searches (*E*-value < 1e−10 and percent_ID >90%) were conducted against ENSEMBL barramundi cDNAs (https://asia.ensembl.org/Lates_calcarifer/Info/Index) using nucleotide sequences of the predicted akame genes as queries. Completeness of the annotated protein-coding gene set was estimated by BUSCO version 5.1.2 (Simão et al. 2015) with a database of ray-finned fishes (Actinopterygii odb10).

### Reconstruction of the Demographic History of Akame and Barramundi

We used PSMC v0.6.5 software (Li and Durbin 2011) to infer the historical *N*_e_s of akame and barramundi. For akame, we used the 10× barcode trimmed Illumina reads of both individuals (Kochi and Miyazaki) sampled in this study. For barramundi, we retrieved whole genome sequencing (WGS) data of individuals of all the three major populations of barramundi collected from the Western coast of India (SRR3183258), Thailand (SRR3183264), and Indonesia (SRR3183270); these individuals were deeply sequenced in a previous low coverage WGS project (Vij et al. 2016). These Illumina reads were aligned to the corresponding repeat masked chromosome-level assembly of akame or barramundi using BWA-MEM v0.7.17 (Li and Durbin 2009) with the default parameters. We subsampled 20% of the mapped reads of akame individuals to equalize coverage across samples, resulting in mean depths of 24 to 33. Variant calling was performed using SAMtools v1.10 mpileup utility (Li 2011) (-q 20, -Q 20, and -C 50), and the FASTQ transformed consensus sequences were generated with vcfutils.pl (vcf2fq -d 10 -D 60). The input file for the PSMC modeling was created using fq2psmcfa tools (-q 20, -g 10000, and -s 10) and processed in psmc program with 25 iterations using the following parameters: -N 25, -t 10, -r 5, -p “4 + 25 * 2 + 4 + 6.” In order to prevent overfitting, we confirmed the inference of at least 10 recombinations in each time interval after 20 rounds of iterations with these parameter settings. Bootstrapping (100 replicates) was performed by random sampling with the replacement of 50-kb sequence segments generated by splitfa scripts. For scaling the inferred population history, we used the same parameters as used in the previous demographic analysis of the barramundi (Wang et al. 2016), namely a generation time of 4 years (Jerry 2013) and a mutation rate of 1.0 × 10^−8^ substitutions per year (Brumfield et al. 2003).

### Genotyping and Functional Annotation of SNVs

To assess the genetic diversity in the akame genome, 10× barcode-trimmed Illumina reads of Kochi and Miyazaki individuals were mapped to the chromosome-level assembly (Kochi individual). The main steps for SNV calling and genotyping are given in supplementary fig. S11, Supplementary Material online. First, paired-end Illumina reads were cleaned by filtering adaptor sequences and trimming low-quality bases using FaQCs (Lo and Chain 2014). Second, in each individual, the cleaned reads were mapped to the reference genome by NextGenMap (Sedlazeck et al. 2013), and a BAM file was generated. The BAM file was sorted by SAMtools and mapping quality was checked by QualiMap program package (García-Alcalde et al. 2012). In the sorted BAM file, PCR duplicates were marked using the Picard toolkit (http://broadinstitute.github.io/picard/) and local realignments of INDELs were conducted by GATK v3.8.1 (McKenna et al. 2010). In the BAM file for each individual, GVCF files were generated using GATK HaplotypeCaller tool with options -hets 0.001 and -indelHeterozygosity 0.001. Finally, GVCF files for the two individuals were jointly genotyped and an output VCF file was generated using GATK GenotypeGVCFs tool. For genotyped SNVs, variant filtering was applied using GATK VariantFilteration tool with the following cutoff values: MQ > 30.00, SOR < 4.000, QD > 2.00, FS < 60.000, MQRankSum > −20.000, ReadPosRankSum > −10.000, and ReadPosRankSum < 10.000.

Genome-wide empirical single nucleotide polymorphism (SNP) heterozygosity in the two akame genomes calculated the fraction of heterozygous SNVs in all effective nucleotide sites in the draft genome (=genome effective length: Total genome length − number of N sites). To assess the difference in genetic diversity between akame and barramundi, the genome-wide SNP heterozygosity in the individual of barramundi from SG, the genome of which has been deeply sequenced (*ca.* 80× coverage; Vij et al. 2016), was also estimated in the same way. Illumina paired-end reads (accession nos. SRR3140997 and SRR3140998) of the SG individual were mapped to the barramundi reference genome assembly, and the number of heterozygote SNV sites was counted.

To assess the genetic load in akame and barramundi, the ratio of the heterozygosity of 0-fold (the proxy for nonsynonymous sites) and 4-fold (the proxy for synonymous sites) degenerate sites in protein-coding regions were calculated in the two akame (Kochi and Miyazaki) and four barramundi (SG, India, Thailand, and Indonesia) individuals, using the similar method as Robinson et al. (2016) and Robinson et al. (2018). SNVs in barramundi individuals were identified by mapping their Illumina reads to the chromosome-level assembly of the akame (Kochi) genome, applying the same filtering criteria in the SNV calling of the two akame individuals. Total numbers and positions of 0- and 4-fold degenerate sites were counted using DEGENOTATE program (Mirchandani et al. 2024). In each individual of akame and barramundi, heterozygous 0- and 4-fold degenerate sites were retrieved using BEDTools (Quinlan and Hall 2010). In addition, functional effect categories of SNVs (High: Start lost, stop gained, and splice acceptor/donor variants; Moderate: Missense variants; Low: Synonymous, stop retained, etc.) in the protein-coding regions were also estimated in these individuals. Variant annotation and functional effect categorization of SNVs in the akame and barramundi genomes were performed using the SnpEff program (Cingolani et al. 2012), based on the predicted gene set of the Akame (Kochi) genome. In this analysis, only heterozygous SNVs were counted and classified into the functional categories. The protein-coding SNVs in akame and barramundi were also classified into “deleterious” and “tolerated” functional categories by SIFT missense prediction method (Vaser et al. 2016). Initially, a custom SIFT database of the akame genes was generated using Swiss-Prot protein sequences downloaded by UniProt database (https://www.uniprot.org). Next, functional categories of SNVs in each individual of akame and barramundi were inferred using the SIFT 4G program. In SIFT missense prediction, both homozygote and heterozygote SNVs were considered and counted separately.

### Runs of Homozygosity

In Kochi and Miyazaki individuals of akame and the SG individual of barramundi, ROHs were called using the “bcftools roh” command in BCFTools version 1.7 (Li 2011), with the default allele frequency value set at 0.4 (-AF-dflt 0.4). Indels were not included for the detection of ROH. *F*_ROH_ was calculated as the length of the genome within ROH of at least 100 kb divided by the total length of the genome (chromosome-level assembly).

### Nucleotide Diversity-Peak Analysis

A sliding-window analysis of nucleotide diversity was carried out to examine the genome-wide distribution of polymorphic sites in the akame genome. The nucleotide diversity of the akame genome was calculated by passing filters in nonoverlapping 10-kb windows, using the SNVs obtained from the genome sequences of the Kochi and Miyazaki individuals. Nucleotide diversity in each window was calculated using the window-pi program implemented in the VCFtools software package (Danecek et al. 2011). Some genomic regions with unusually high nucleotide diversity may be associated with unannotated repeats or segmental duplications. Thus, on the same sliding-window interval used for nucleotide diversity, estimation of the mappability scores and the depth of coverages were also conducted, and windows with unusual values were excluded from the analysis as possible artifacts. The mappability scores were estimated by GenMap (Pockrandt et al. 2020). Windows with mappability of <0.8 were excluded from the nucleotide diversity-peak analysis. The depth of coverage in each window was estimated by TinyCov (https://github.com/cmdoret/tinycov). Windows with unusually low (<40×) and high (>400×) mean coverages were also excluded from the analysis. Peaks were identified as windows with nucleotide diversity in excess of 4 SDs above the mean across the genome. Enrichment analysis was performed on genes within the nucleotide diversity peak regions using g:Profiler (Raudvere et al. 2019) with the ENSEMBL barramundi perch annotation (Vij et al. 2016). We defined the genes with SNP heterozygosity >0.01 in the protein-coding region located within the nucleotide diversity peak regions as “polymorphic genes” and indicated in the window plot (see Fig. 4a and Table 2). Genes in the nucleotide diversity-peak regions were also classified by functional annotation clustering using DAVID web server (https://david.ncifcrf.gov/gene2gene.jsp; Sherman et al. 2022).

To quantify the genome-wide distribution of nucleotide divergence between akame and barramundi, a sliding-window analysis of *F*_ST_ between akame and barramundi was carried out. Mean *F*_ST_ values between akame (Kochi and Miyazaki) and barramundi (SG, India, Thailand, and Indonesia) were estimated by passing filter in nonoverlapping 10-kb windows, similar to the estimation of nucleotide diversity. Initially, in each of the akame and barramundi individuals, Illumina reads were mapped to the chromosome-level assembly of the akame (Kochi) genome. After indel realignments, GVCF files were generated using GATK HaplotypeCaller tool with options -hets 0.001 and -indelHeterozygosity 0.001. GVCF files for the six individuals were jointly genotyped and an output VCF file was generated using GATK GenotypeGVCFs tool. Finally, variant filtering was applied using the GATK VariantFilteration tool, using the same criteria in the SNV calling of the two akame individuals. The mean *F*_ST_s in each window were calculated using the weir-fst-pop program implemented in the VCFtools software package (Danecek et al. 2011). Nucleotide diversity per 10 kb nonoverlapping window in barramundi was also calculated from the four individuals using the window-pi program implemented in the VCFtools software package (Danecek et al. 2011). Nucleotide diversity-peaks were identified as windows with nucleotide diversity in excess of 4 SDs above the mean across the genome. Windows of the nucleotide diversity-peaks common in akame and barramundi were detected by comparing the results of the sliding-window analysis between the two species. To test whether the number of the observed common nucleotide diversity-peak windows was significantly larger than that expected by chance, the reference distribution of the numbers of windows common to the two species under the random expectation was generated using the Monte Carlo method of random sampling with 1,000,000 replicates. *P*-value was calculated as the proportion of trials that show a larger number of common windows than observed. The R source code of the Monte Carlo simulation was provided in supplementary appendix, Supplementary Material online.

### Analysis of Selective Forces on Akame and Barramundi Genes

To assess selective forces on akame genes by molecular evolution-based analysis, orthologous relationships between genes of akame (Kochi individual) and other fish species were inferred based on homology and species phylogeny. First, transcript sequences of barramundi, *Oryzias latipes* (Japanese medaka), *Perca fluviatilis* (European perch), and *Hippocampus comes* (tiger tail seahorse) were downloaded from the NCBI database. Second, protein-coding sequences of these transcripts were predicted by TransDecoder (https://github.com/TransDecoder/TransDecoder/wiki). Third, orthology relationships were inferred by all-by-all BLAST, Markov clustering, and phylogenetic tree inference implemented in the Python programs by Yang and Smith (2014). Codon-based nucleotide sequence alignment was constructed in each of the orthologous gene datasets obtained from the one-to-one (homologs without any taxon repeat) and the MI (an orthology inference method that iteratively cuts out the subtree with the highest number of taxa without taxon duplication) algorithms. Initially, protein sequences for each of the ortholog sequences were aligned by MAFFT version 7 (Katoh and Standley 2013). Next, a codon-based nucleotide sequence alignment was generated using a custom Perl script, by referring to the protein alignment. Then, the phylogenetic relationship of the five species was reconstructed by using the concatenated dataset of all one-to-one orthologs, generated by Phyutility software (Smith and Dunn 2008). A maximum-likelihood tree (supplementary fig. S8, Supplementary Material online) was reconstructed by the multithreading version of RAxML program (Stamataxis 2014) and the reliability of each tree node was assessed by 1,000 replications of the rapid-bootstrap method implemented in RAxML. Topology of the maximum-likelihood tree was used for the evolutionary analyses described below.

To infer the changes in selective pressure to the genes in akame or barramundi, a selection intensity parameter (*k*) was inferred using RELAX descriptive models (Wertheim et al. 2015) in all one-to-one and MI ortholog gene sets in the five species. In RELAX analysis, a branch connecting to akame or barramundi was set to “test branch” and other branches were considered to the reference branches. Model parameters (three categories of *ω* = *d*_N_/*d*_S_ values and their proportions in test and reference branches) were estimated in null (*k* was constrained to 1) and alternative (*k* was a free parameter, *k* ≥ 0) models, and a hypothesis for relaxation or intensification of selection is tested by an LRT using the standard *χ*^2^ distribution with one degree of freedom. In each of the two species, the numbers of genes under relaxed functional constraints (*k* < 1) and intensified selective pressure (*k* > 1; i.e. positive or purifying selection) were counted. The analysis was conducted using the RELAX program implemented in the HYPHY-MPI software package (Pond et al. 2005) and the results were retrieved using a custom Python script.

In addition, differences in the evolutionary rates between genes in akame and barramundi were tested by estimating nonsynonymous–synonymous substitution rate ratios (*ω* = *d*_N_/*d*_S_) in all one-to-one and MI ortholog gene sets. Nonsynonymous–synonymous substitution rate ratios of the genes along the tree for these species were estimated by the maximum likelihood-based method developed by Yang (1998), using the CODEML program in the PAML 4.7a software package (Yang 2007). Nonsynonymous–synonymous substitution rate ratios for each tree branch were estimated under the branch-specific models of codon evolution (Yang 1998). Details of the models for *ω* estimation used in this study are described in the “Results” section. GO enrichment analysis was performed on genes that showed *ω* > 1 with FDR-adjusted *P* < 0.05 in the log-likelihood test in akame or barramundi using g:Profiler (Raudvere et al. 2019) with the ENSEMBL barramundi annotation.

## Supplementary Material

evae174_Supplementary_Data

## Data Availability

The scaffold-level genome assemblies in akame (Kochi: BRZM01000000, Miyazaki: BAABXA010000000) have been deposited in the DNA Data Bank of Japan (DDBJ) genome database. The Chromium-linked Illumina reads, RNA-Seq reads (Illumina HiSeq), and Nanopore reads utilized for the genome assembly have been deposited to the DDBJ sequence read archive (DRA) under the BioProject accession number PRJDB13763. The chromosome-level assembly and the GFF3 annotation files of akame (Kochi) were deposited in Dryad (URL: https://doi.org/10.5061/dryad.m37pvmdb6).
