# Supplementary material for: Draft Genome of Akame (Lates Japonicus) Reveals Possible Genetic Mechanisms for Long-Term Persistence and Adaptive Evolution with Low Genetic Diversity
Source: Genome Biol Evol. 2024 Aug 7;16(8):evae174. doi: 10.1093/gbe/evae174 (PMC11346364; doi:10.1093/gbe/evae174)
Supplement: evae174_Supplementary_Data [file evae174_supplementary_data.zip › Supplementary_Materials_240714.pdf]

Supplementary Figures and Appendix

Draft genome of akame (*Lates japonicus*) reveals possible genetic mechanisms for long-term persistence and adaptive evolution with low genetic diversity

Yasuyuki Hashiguchi, Tappei Mishina, Hirohiko Takeshima, Kouji Nakayama, Hideaki Tanoue, Naohiko Takeshita, Hiroshi Takahashi

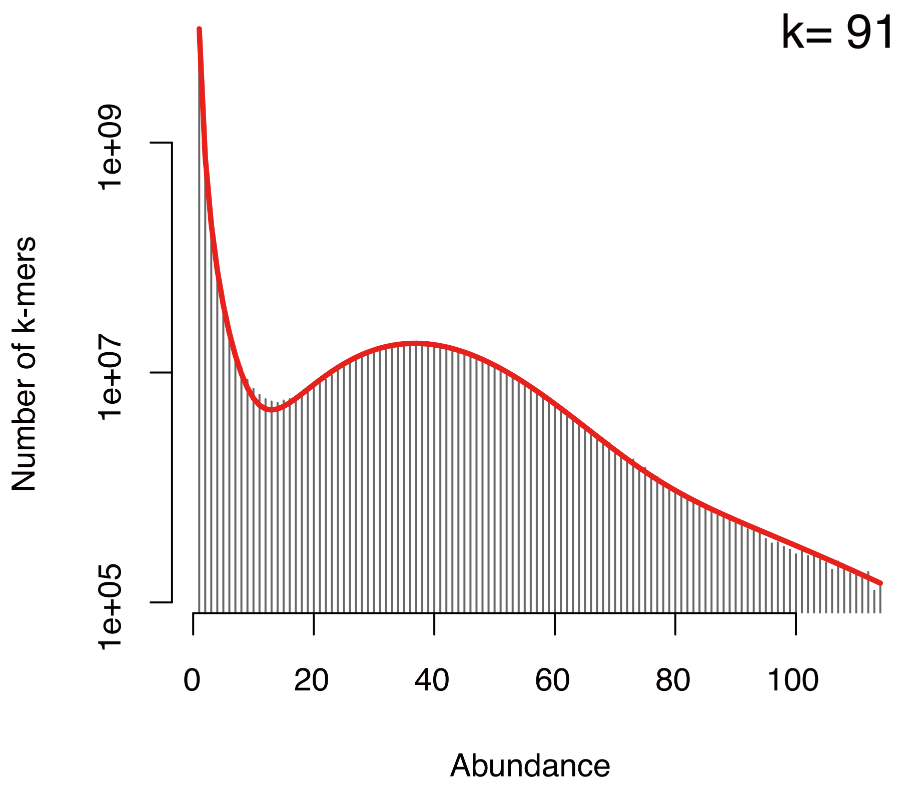

Fig. S1. Histogram of the optimal k-mer size (k = 91) in the akame (Kochi) genome. Red line indicates the fitting curve fitted using the complete statistical model of the histogram (erroneous k-mers + genomic k-mers).

Akeme (Kochi genome)

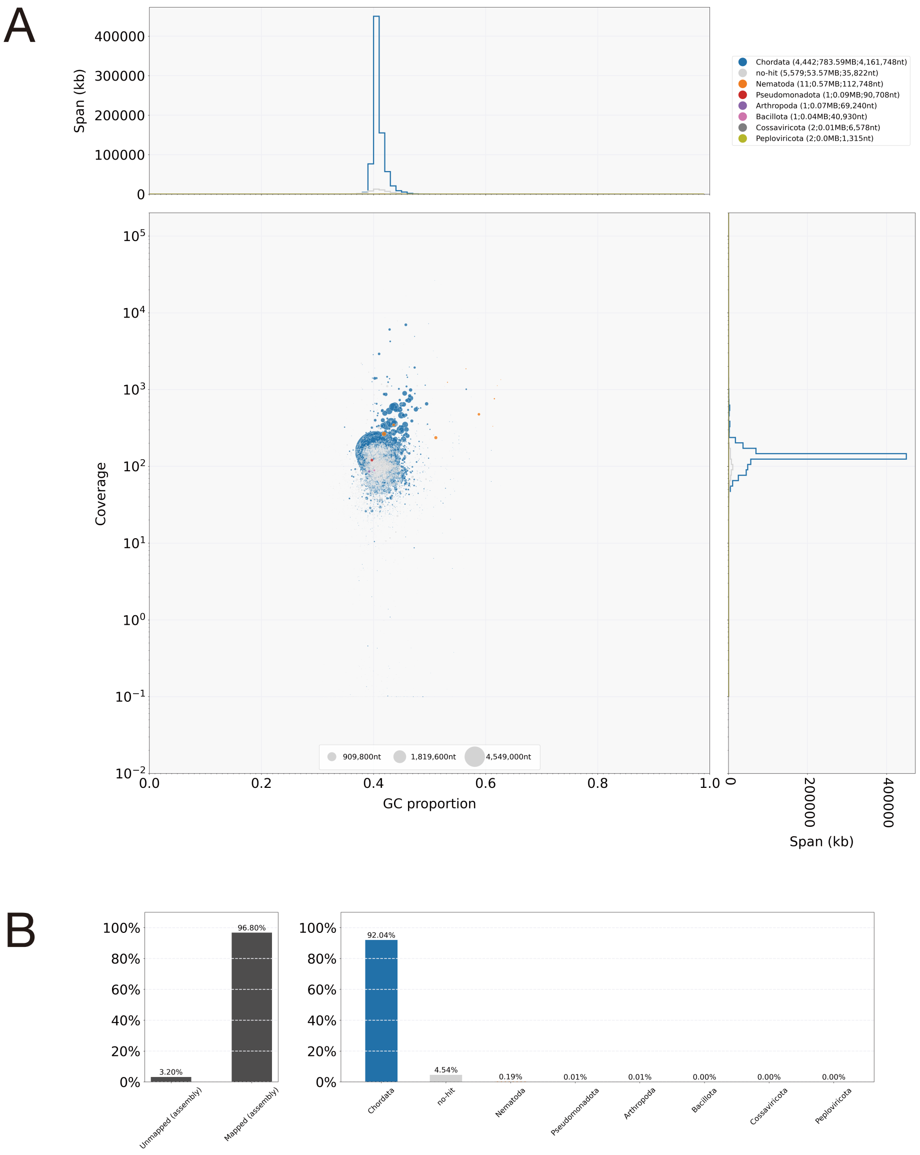

Fig. S2. Summary of BlobTools analysis of the akame (Kochi) genome. **A**: Blobplot (a scatter plot of GC proportion and coverage in each scaffold, decorated with coverage and GC histograms) of the scaffold-level assembly of the akame (Kochi) genome. **B**: Proportions of the sequences of organisms contained in the scaffold-level assembly of the akame (Kochi) genome.

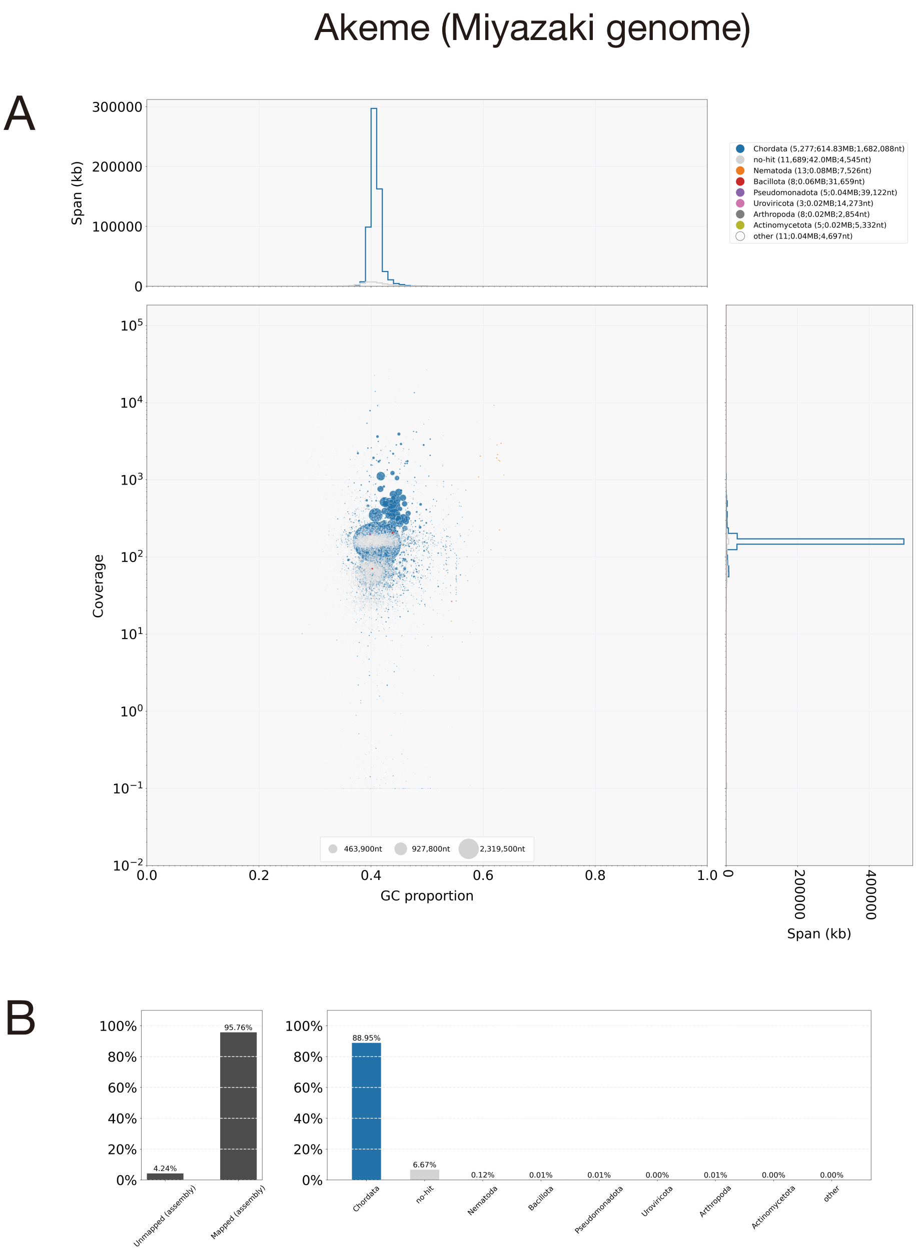

Fig. S3. Summary of BlobTools analysis of the akame (Miyazaki) genome. **A**: Blobplot of the akame (Miyazaki) genome assembly. **B**: Proportions of the sequences of organisms contained in the s akame (Miyazaki) genome assembly.

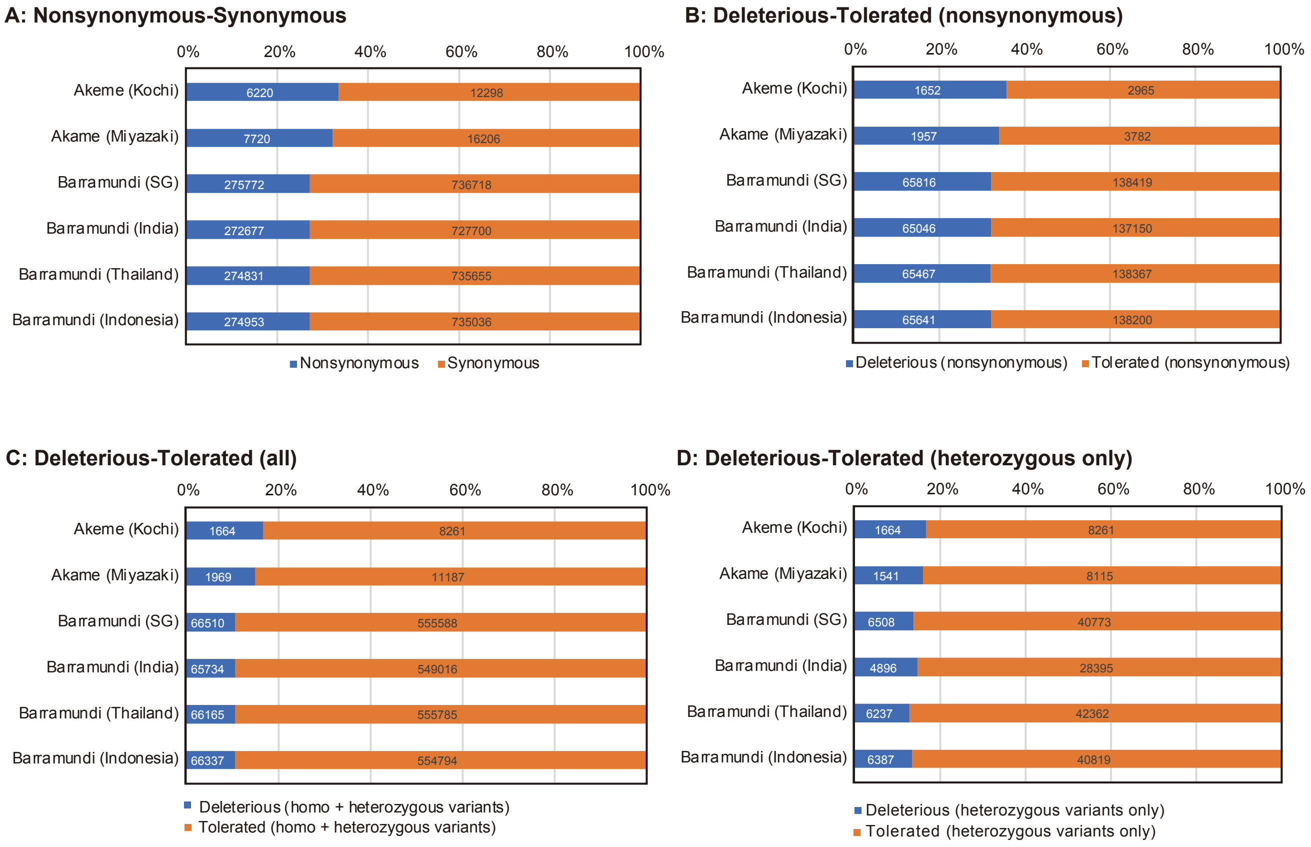

Fig. S4. Summary of the SIFT missense prediction in the single nucleotide variants of the akame and barramundi genes. Numbers of variants are shown in the bars. **A**: The proportions of nonsynonymous and synonymous variants (homozygous + heterozygous variants). **B**: The proportions of "deleterious" and "tolerated" missense variants. **C**: The proportions of deleterious and tolerated variants (including synonymous variants). **D**: The proportions of deleterious and tolerated variants (heterozygous variants only).

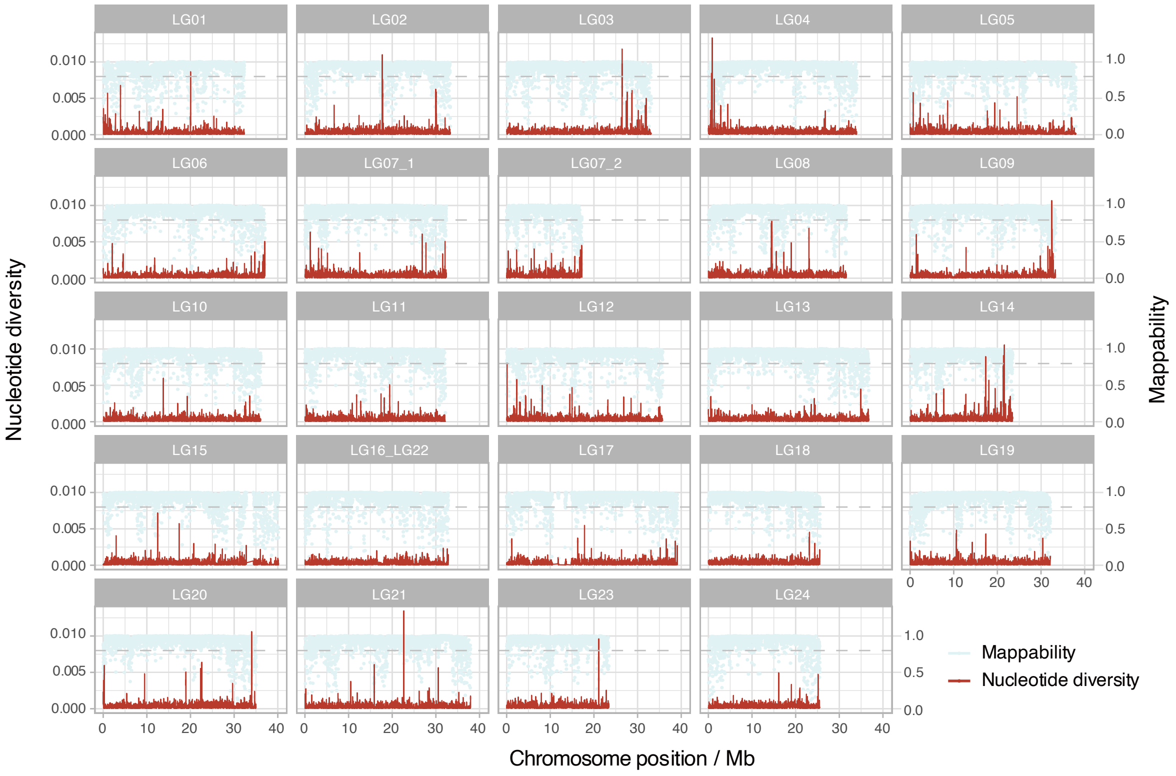

Fig. S5. A sliding-window plot of nucleotide diversity (red: left y-axis) and mappability score (light blue: right y-axis) for 10-kb non-overlapping windows in the akame genome. The mitochondrial genome was not included in the analysis. The mean mappability score in the akame (Kochi) genome was 0.91. Windows of low mappability (<0.8; shown by dotted line) were excluded from genome-wide nucleotide diversity analysis.

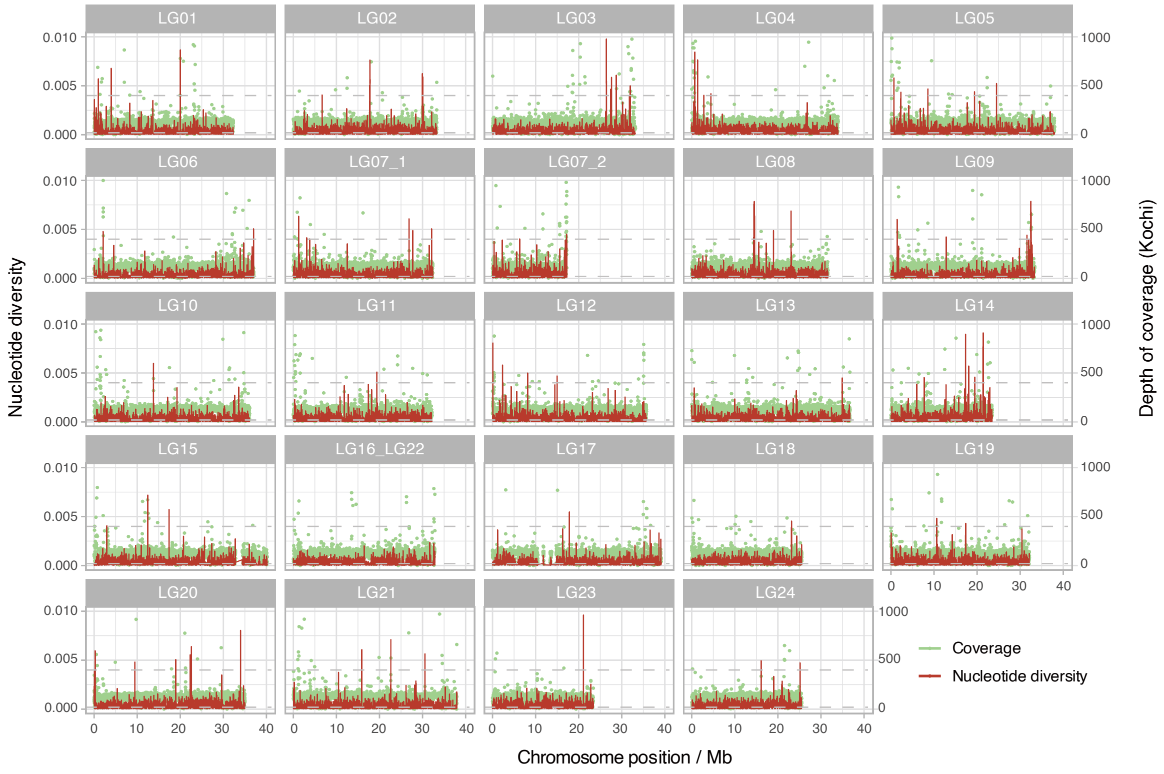

Fig. S6. A sliding-window plot of nucleotide diversity (red: left y-axis) and mean depth of coverage (green: right y-axis) for 10-kb non-overlapping windows in the akame genome. The mitochondrial genome was not included in the analysis. The mean depth of coverage in the akame (Kochi) genome was 129.1, and the windows of unusually high (>400) or low (<40) depth of coverage (shown by dotted line) were excluded from the analysis of the genome-wide nucleotide diversity.

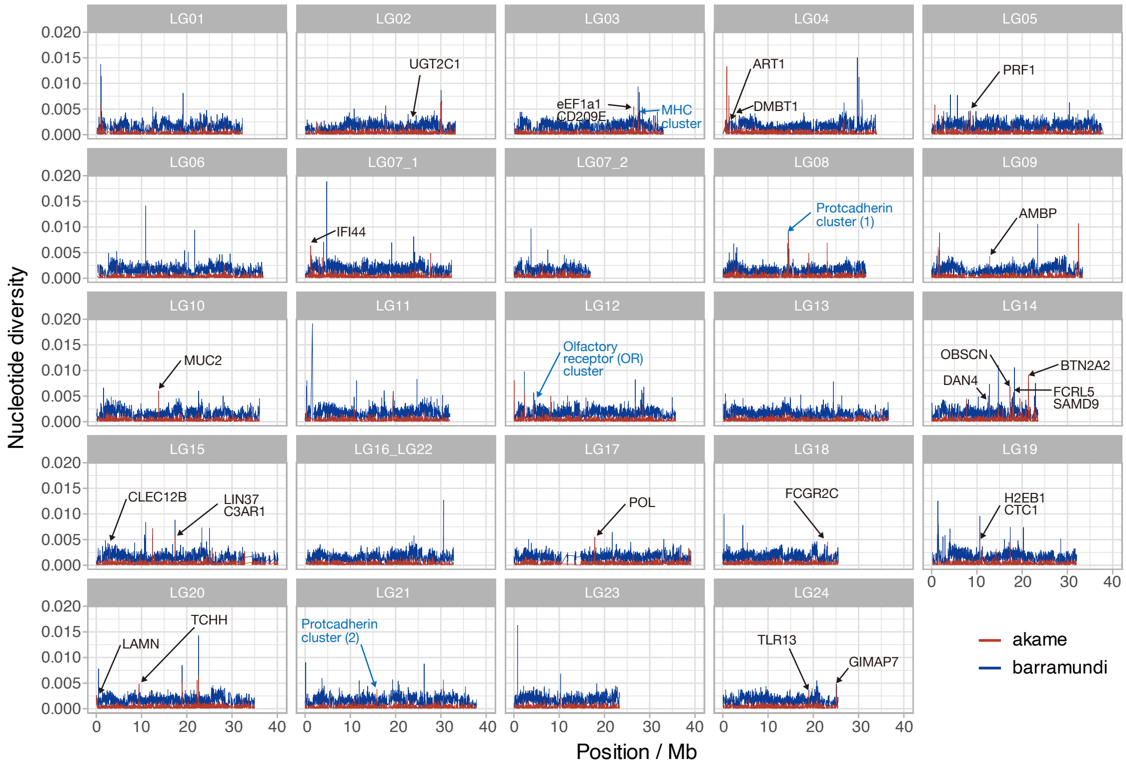

Fig. S7. A sliding-window plot of nucleotide diversity in the akame (red) and barramundi (blue) genomes for 10-kb non-overlapping windows. The mitochondrial genome was excluded from the analysis. Abbreviations of the representative polymorphic genes in the nucleotide diversity peak regions (nucleotide diversity within the coding region > 0.01) are shown in the figure (see Fig. 4A and Table 2 for details). The genomic locations of the MHC, olfactory receptor, and two protocadherin gene clusters within the nucleotide diversity-peak regions are indicated with blue arrows.

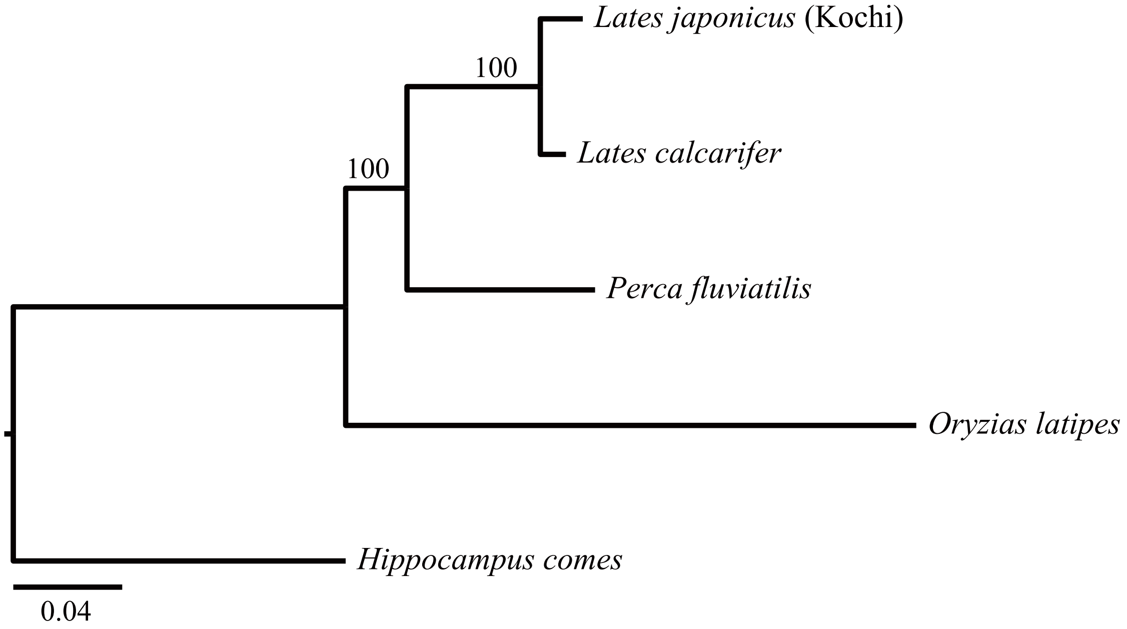

Fig. S8. Maximum likelihood phylogenetic tree based on 6,847 one-to-one ortholog genes in the five fishes. All genes (>300 bp) were concatenated using Phyutility (Smith and Dunn 2008), and the ML tree was reconstructed by RAxML (Stamataxis 2014) with the GTRCAT + Γ model. Reliability of the tree nodes was assessed by the rapid-bootstrap method with 1,000 replications, implemented in RAxML. Seahorse was selected as an outgroup species on the basis of the phylogenetic relationship presented in Hughes et al. (2018).

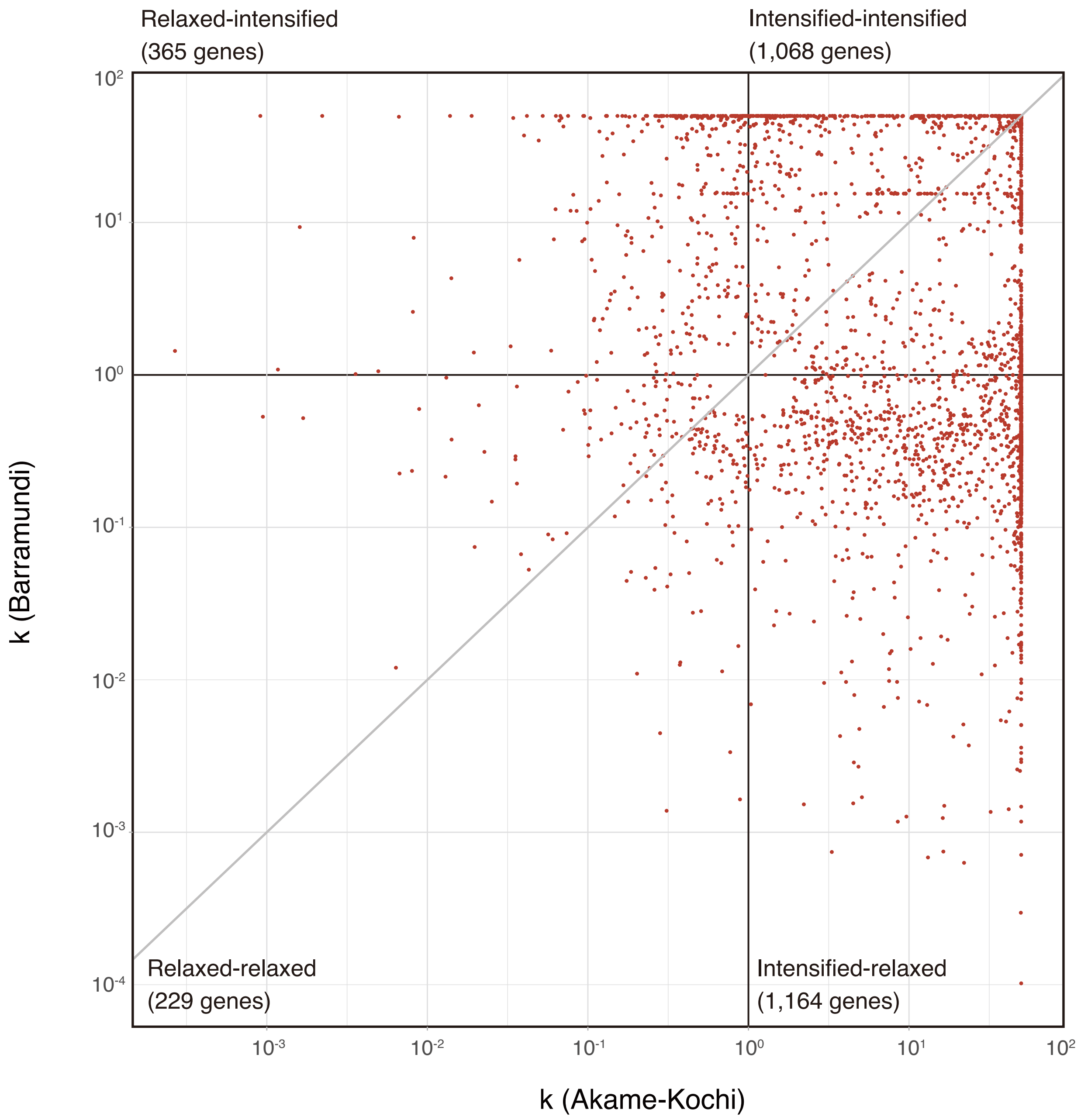

Fig. S9. Distribution of the relaxation parameter (*k*) in 2,828 akame and barramundi genes showing intensified or relaxed selections were detected in one or both species ($P < 0.05$). For simplicity, this figure does not include 122 genes that had *k* values of $<10^{-4}$. Diagonal line indicates that *k* in akame (Kochi) equals to that in Barramundi.

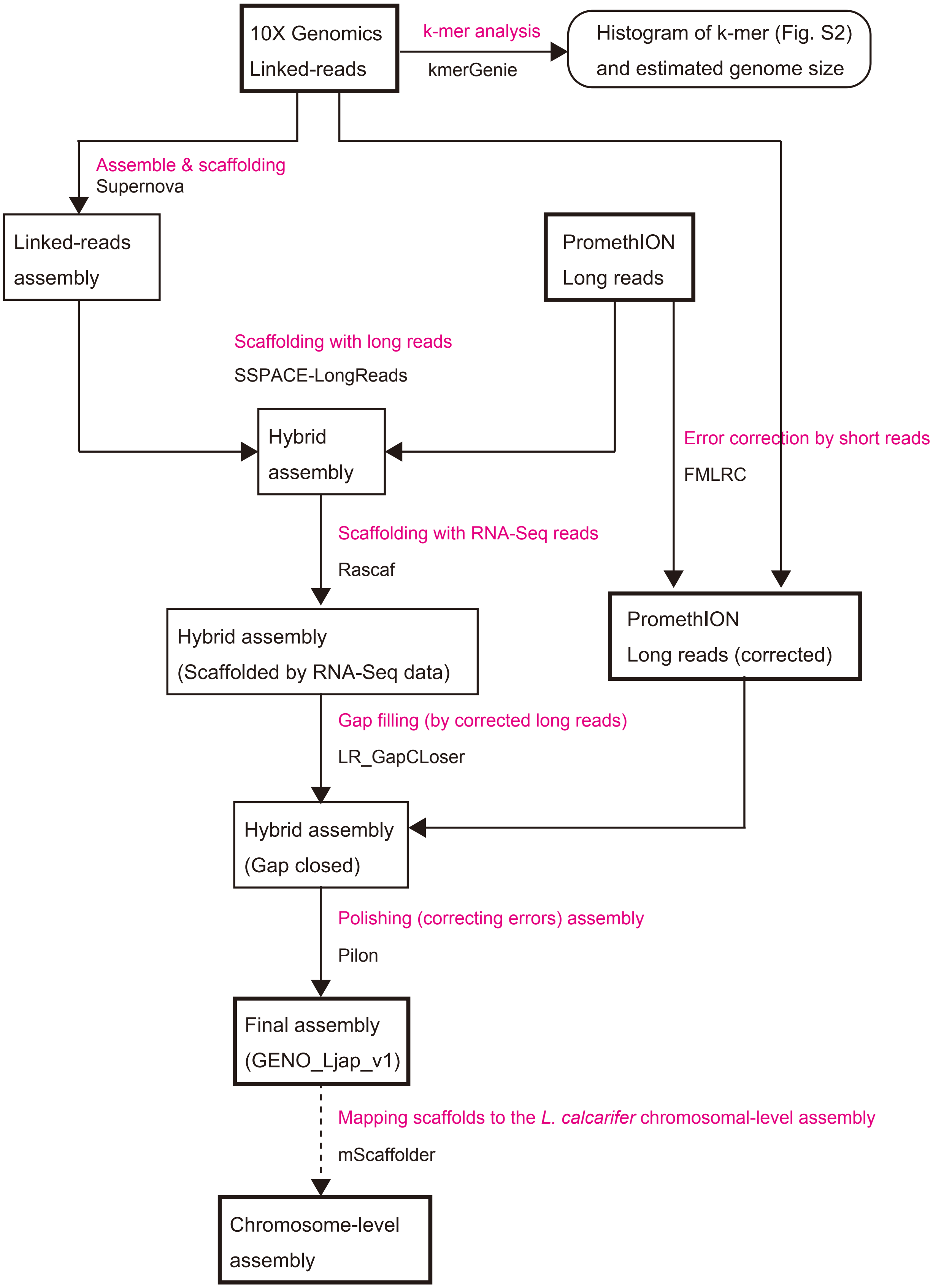

Fig. S10. A flowchart of the stages in the assembly of the akame reference genome (Kochi).

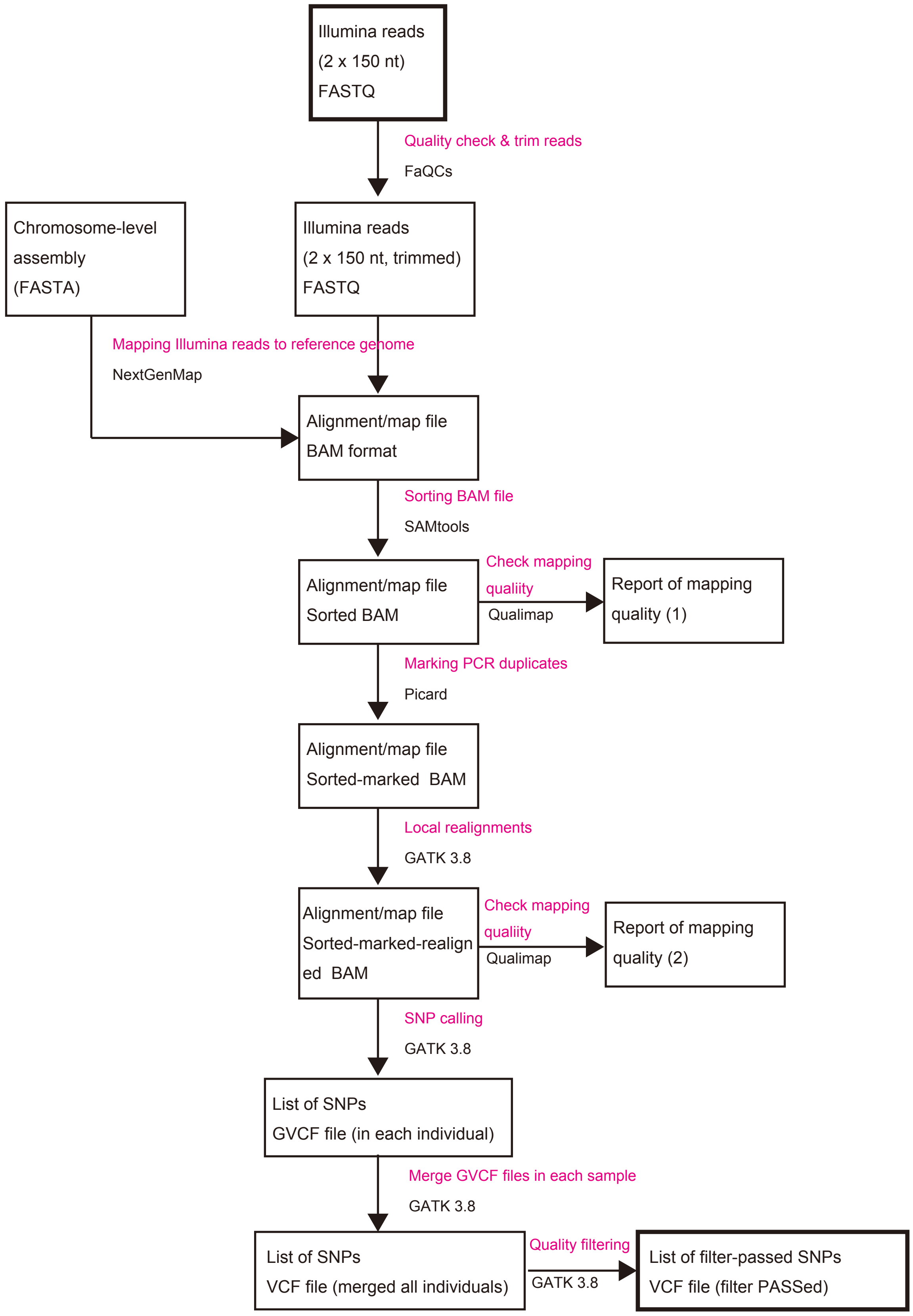

Fig. S11. A flowchart of the analyses for variant detection, SNV genotyping, and functional annotation of the akame genomes.

Appendix. The `R` source code of the Monte Carlo simulation for generating the distribution of the high nucleotide diversity (mean + 4-SD) windows common to the akame and barramundi genomes.

```
# Monte Carlo simulation by heterozygosity peaks

# number of common window
common_peak <- 44

# simulation
# setting
n.peak.akame <- 230
n.peak.barramundi <- 131
n.effective.windows.akame <- 52459
n.effective.windows.barramundi <- 52459
n.simulation <- 1000000

# main
result <- NULL
for (i in 1:n.simulation){
  akame.window.i <- sample(1:n.effective.windows.akame, n.peak.akame)
  barramundi.window.i   <-   sample(1:n.effective.windows.barramundi,
n.peak.barramundi)
  result.i <- sum(akame.window.i %in% barramundi.window.i)
  result <- c(result, result.i)
}

# p-value
condition <- (result > common_peak)
large_common <- result[condition]
pval <- length(large_common)/length(result)

# histogram of the simulation result
hist(result,breaks=seq(0,10,by=1))
```
